# Bond-centric modular design of protein assemblies

**DOI:** 10.1038/s41563-025-02297-5

**Published:** 2025-07-31

**Authors:** Shunzhi Wang, Andrew Favor, Ryan D. Kibler, Joshua M. Lubner, Andrew J. Borst, Nicolas Coudray, Rachel L. Redler, Huat Thart Chiang, William Sheffler, Yang Hsia, Neville P. Bethel, Zhe Li, Damian C. Ekiert, Gira Bhabha, Lilo D. Pozzo, David Baker

**Affiliations:** 1 Department of Biochemistry, University of Washington, Seattle, WA USA; 2 Institute for Protein Design, University of Washington, Seattle, WA USA; 3 Molecular Engineering and Sciences Institute, University of Washington, Seattle, WA USA; 4 Department of Biology, Johns Hopkins University, Baltimore, MD USA; 5 Department of Medicine, Division of Precision Medicine, NYU Grossman School of Medicine, New York, NY USA; 6 Department of Chemical Engineering, University of Washington, Seattle, WA USA; 7 Howard Hughes Medical Institute, University of Washington, Seattle, WA USA

**Keywords:** Biomaterials - proteins, Protein design

## Abstract

Directional interactions that generate regular coordination geometries are a powerful means of guiding molecular and colloidal self-assembly, but implementing such high-level interactions with proteins remains challenging due to their complex shapes and intricate interface properties. Here we describe a modular approach to protein nanomaterial design inspired by the rich chemical diversity that can be generated from the small number of atomic valencies. We design protein building blocks using deep learning-based generative tools, incorporating regular coordination geometries and tailorable bonding interactions that enable the assembly of diverse closed and open architectures guided by simple geometric principles. Experimental characterization confirms the successful formation of more than 20 multicomponent polyhedral protein cages, two-dimensional arrays and three-dimensional protein lattices, with a high (10%–50%) success rate and electron microscopy data closely matching the corresponding design models. Due to modularity, individual building blocks can assemble with different partners to generate distinct regular assemblies, resulting in an economy of parts and enabling the construction of reconfigurable networks for designer nanomaterials.

## Abstract (web summary)

Deep learning-based generative tools are used to design protein building blocks with well-defined directional bonding interactions, allowing the generation of a variety of scalable protein assemblies from a small set of reusable subunits.

## Main

Bonding is central in chemistry for generating the interactions between atoms in small and large molecules^1^. High structural complexity and designability emerges from a relatively small set of atoms and bonding geometries, enabling the placement of large numbers of atoms at precisely defined distances and orientations with predictable interaction strengths. Such modularity is also critical to stepwise molecular synthesis^2^. Supramolecular systems^3^ based on analogous bonding concepts have been generated with well-defined nanoscale structures, using host–guest^4^, metal coordination^5^ and canonical DNA base-pairing interactions^6^. However, generating protein assemblies using predictable bonding through protein–protein interactions remains challenging due to the complex sequence–structure relationships of proteins and their high folding cooperativity. Overcoming this challenge could enable the creation of precisely engineered protein nanomaterials with broad applications in medicine, synthetic biology and biotechnologies. Precise interface alignment requires sequence optimization on both sides^7,8^, which can impact the overall protein folding and make designing new assemblies non-trivial. Despite advances in deep learning-based computational methods, the robust prediction^9,10^ and design^11,12^ of multicomponent architectures beyond cyclic oligomers^11,13^ continues to be a challenge. In particular, unbounded structures, such as two-dimensional (2D) and three-dimensional (3D) lattices, have only been created by the computational docking^14–17^ of prevalidated building blocks with complementary shapes and moderate experimental success rate. Recently, the WORMS^18^ protocol has been developed to construct symmetric assemblies from predefined cyclic oligomers by the large-scale sampling of possible junction geometries generated by fusing helical repeat protein building blocks. However, the WORMS approach offers limited control over the relative orientation as well as spacings between components, and the generated monomers are often extended, which may lead to off-target assemblies^19^. A strategy that enables the versatile combinatorial construction of complex architectures with prespecified geometries from a small number of rigid building blocks could have considerable advantages in modularity, property predictability and component reconfigurability.

**Fig. 1:**
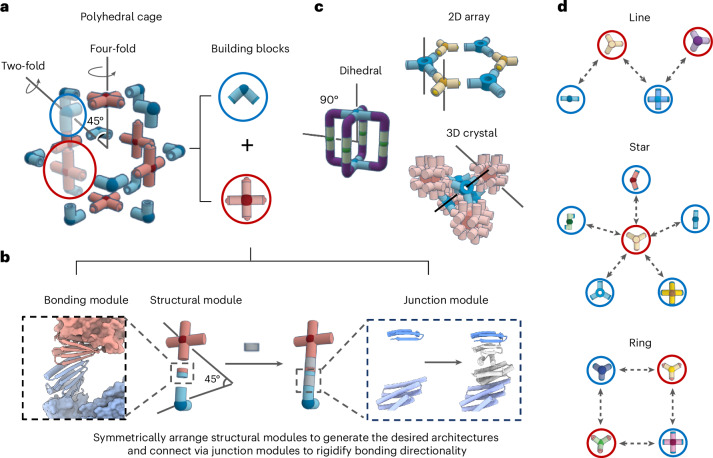
Modular design of bounded and open protein assemblies. **a**, Modular design of protein assemblies based on symmetrically arranged cyclic oligomeric structural modules rigidly connected by bonding modules. **b**, An asymmetric subunit containing structural and bonding modules is positioned to generate the desired architectures. Gaps in between these two modules are connected by rigid junction modules. To ensure bonding directionality, junction modules generated by RFdiffusion or the template-based helical fusion protocol WORMS rigidly bridge between two interfaces. Many alternative backbones can be generated in silico for stabilizing module gaps at the specified orientation (grey junction region). **c**, Schematic of how bounded protein assemblies with dihedral symmetries can be created by controlling the intersecting angle defined by the principal rotational axes. For unbounded structures, cyclic symmetric structural modules are aligned relative to each other according to the space group definitions. **d**, Design of protein–protein interaction networks with distinct topologies: line (top), star (middle) and ring (bottom), mediated by colour-coded complementary bonding modules. Each node represents a de novo designed oligomer, and each edge corresponds to a unique architecture assembled from two adjacent nodes. Source data

We set out to develop a general protocol for designing programmable protein architectures with building blocks that share specific interfaces that fit together to generate a wide diversity of closed and open 3D architectures (Fig. 1). We reasoned that reversible heterodimeric proteins, such as LHDs^20^, could be used as programmable bonding modules on oligomeric building blocks with appropriately matched internal geometry (Fig. 1a). Distinct symmetric architectures could be targeted by aligning pairs of building blocks at specific intersecting angles between their primary rotational axes^21^ (Fig. 1b). Since flexibly grafted bonding modules can lead to ill-defined aggregates or hydrogels^22^, rigid junction adaptors would be required to ensure the precise placement and orientation of individual modules. We reasoned that the wide structural diversity now achievable with de novo protein design could enable the creation of directional bonding geometries beyond those possible with chemical bonds.

LHDs are ideal bonding modules due to their polar yet high-affinity interfaces, with specificity driven by shape complementarity, hydrophobic surface area burial and precisely designed interfacial hydrogen bonds. We used a three-step computational approach to explore the building of protein assemblies using the predefined protein bonds based on a set of LHD heterodimers. In the first step, we define the overall architecture by selecting the bonding modules (LHDs), the structural modules (often symmetric homo-oligomeric cores) and the degrees of freedom to be sampled. These components are then spatially arranged, leaving gaps between the termini of the structural and bonding modules. In the second step, we generate rigid junction modules between homo-oligomeric cores and LHDs that hold them in the desired relative orientations. For symmetric assemblies, only unique junctions within an asymmetric unit need to be explicitly generated (Fig. 1b). Next, we perform backbone sampling to search for a backbone arrangement to stabilize the target new junction module. For this, we used WORMS^18^ to combine the predesigned helical structural modules for generating the required geometries, or RFdiffusion^11^, a deep generative neural network, to directly create backbones that rigidly link the core and bonding modules. In the third step, we design sequences (Methods) for the newly generated backbone segments and the immediately neighbouring positions. For experimental characterization, we select the designed sequences predicted by AlphaFold2 (ref. ^23^) to fold and assemble as intended. The WORMS approach generally results in extended structures, whereas RFdiffusion excels at creating more compact structures that are favourable for designing 2D arrays and 3D lattices (Fig. 1c). The use of shared bonding modules enables multiple partners to be designed to co-assemble with a single shared building block, forming protein–protein interaction networks with distinct topologies (Fig. 1d).

## Design of binary assemblies using cyclic building blocks

We first tested the approach by designing two-component polyhedral cages from cyclic building blocks. For such structures, the geometric requirement is that the cyclic symmetry axes of the building blocks intersect at predefined angles (for example, the *C*_2_ and *C*_4_ axes form a 45° angle in octahedral assemblies) to ensure proper cage closure. We generated two-component cages (Fig. 1 outlines the strategy) using 12 previously designed *C*_2_, *C*_3_ and *C*_4_ cyclic oligomers and the soluble tightly binding LHD 101 bonding module^20^.

**Fig. 2:**
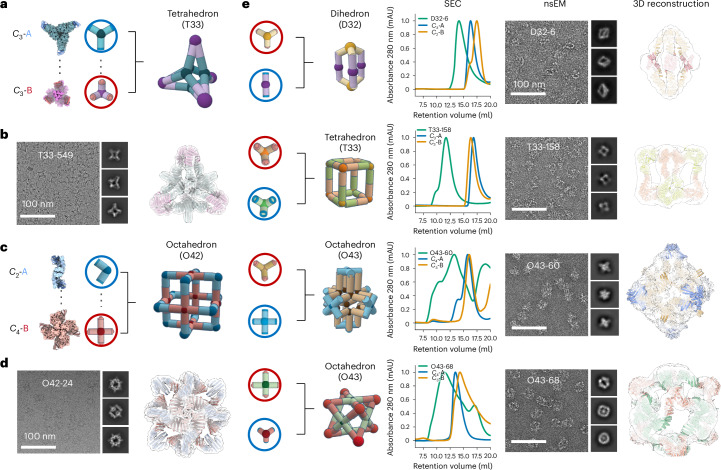
Characterization of designed binary assemblies using cyclic building blocks. **a**, Cartoon of the T33-549 cage assembled from two trimeric building blocks with complementary bonding modules. mAU, milli-absorbance unit. **b**, Representative cryo-EM micrograph of T33-549 cage and 2D class averages (left). The design model fits as a rigid body into the cryo-EM density, showing a close agreement between the design model and cryo-EM reconstruction (right). **c**, Cartoon of the O42-24 cage assembly based on dimeric (blue) and tetrameric (red) building blocks with complementary bonding modules. **d**, Representative cryo-EM micrograph of O42-24 cage and 2D class averages (left). The 3D cryo-EM reconstruction indicates a close agreement with the design model (right). **e**, Structural characterization of four selected assemblies: D32-6, T33-158, O43-60 and O43-68. From left to right: cartoons of the designed assembly from oligomeric building block combinations, SEC elution profiles, structural characterization with nsEM with representative 2D class averages on the side and 3D reconstruction overlaid with the design model. mAU, milli-absorbance unit. Source data

We selected 64 two-component cage designs for experimental characterization with dihedral, tetrahedral and octahedral symmetries. We refer to the designs using the nomenclature [sym]*ab*-*c* (for example O42-24), where *a* and *b* denote the rotational symmetries of the two building blocks, and *c* is a unique design identifier. The selected designs were expressed in *Escherichia coli* using a bicistronic expression system that encodes one of the two building blocks with a C-terminal polyhistidine tag^18^. Complex formation was initially assessed using nickel affinity chromatography, with promising designs showing bands for both building blocks in sodium dodecyl sulfate–polyacrylamide gel electrophoresis after Ni-NTA pulldown. Of the 64 tested designs, 37 passed the bicistronic screen and were selected for individual expression and Ni-NTA purification followed by size exclusion chromatography (SEC). Complexes were assembled in vitro by mixing the SEC-purified components at equimolar ratios. Two cage assemblies were structurally characterized by cryogenic electron microscopy (cryo-EM), yielding 6.1-Å- and an 8.3-Å-resolution reconstructions for designs T33-549 (Fig. 2a,b) and O42-24 (Fig. 2c,d), respectively. The experimental maps are very close to the design models, including the fusion junction region near the heterodimeric bonding module (Supplementary Figs. 1–3). We used negative-stain electron microscopy (nsEM) to characterize the remaining two-component cage designs and identified 3 dihedral, 3 tetrahedral and 5 octahedral designs that assembled into the target cage structures (Fig. 2e). SEC elution profiles indicated that all the constructs could be separately purified as soluble oligomeric building blocks, and readily form cages on in vitro mixing with its partner (Fig. 2e). The most common failure modes were heterogeneous particles, including partial assemblies and soluble aggregates.

## Design of interacting nanomaterial networks

Native proteins can assemble with distinct partners into different oligomerization states that modulate different signalling pathways, for example calmodulin-dependent protein kinase II (ref. ^24^) and Bcl-family proteins^25^. In most de novo designed multicomponent assemblies, the partners are specifically engineered to interact with each other, and hence, interactions with other components are unlikely to form productive complexes^20^.

**Fig. 3:**
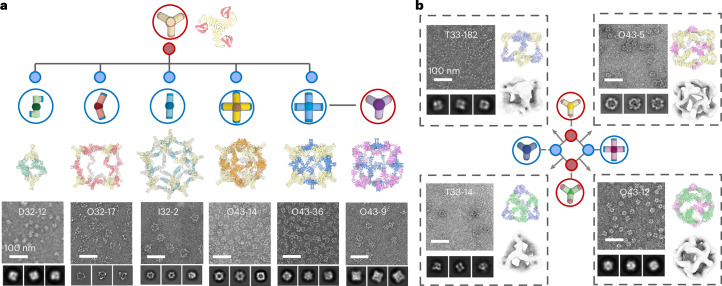
Shareable building blocks enable expansion of binary assembly networks. **a**, Starting from the design model of a *C*_3_ cyclic oligomer with the bonding motif, we generated five distinct complementary assembly partners that generate different closed architectures. From left to right: D32-12 (dimeric partner no. 1), O32-17 (dimeric partner no. 2), I32-2 (dimeric partner no. 3), O43-14 (tetrameric partner no. 4) and O43-36 (tetrameric partner no. 5). nsEM (bottom) single-particle views, 2D class averages and 3D reconstruction are consistent with the design models. We use the same procedure recursively to generate building blocks complementary to the newly designed components: the O43-9 cage (far right) is derived from the secondary *C*_4_ component of the O43-36 assembly. **b**, Group of four building blocks sharing complementary interfaces were designed such that each building block can assemble with two others to form two tetrahedral (T33-182 and T33-14) and two octahedral (O43-5 and O43-12) cages. nsEM indicates homogeneous particles with shapes matching the design models. Scale bar, 100 nm. Source data

**Extended Data Fig. 1:**
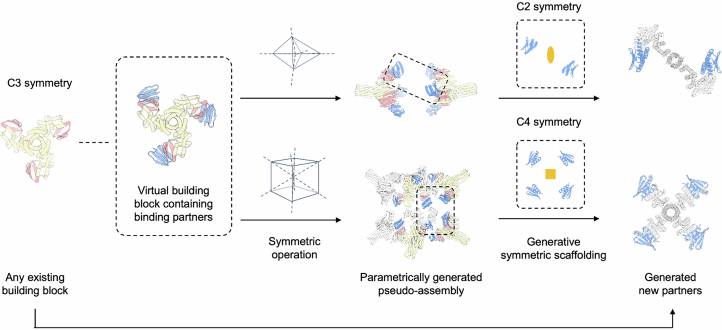
Computational design approach for generating binding partners through symmetric expansion. Our method allows the creation of virtual building blocks, such as C3-AB′, by symmetrically arranging fragments of complementary binding partners for an existing cyclic oligomer, C3-A. The outward-facing virtual building block, C3-AB′, can then be symmetrically arranged to explore rotations and translations along its new symmetry axes within the target assembly. New oligomeric binding partners are isolated and designed using symmetric RFdiffusion or WORMS, enabling the generation of new assembly partners.

We sought to design multiple binding partners that co-assemble with one building block, via the shared bonding modules, forming interacting networks with star, line or ring topologies. To create the star topology, we started with one building block and generated a set of alternative building blocks, leading to distinct assemblies using a slightly modified version of the procedure described above (Fig. 3a, Extended Data Fig. 1 and Methods). We selected a trimeric *C*_3_ building block, *C*_3_-36B, and designed five new *C*_2_ or *C*_5_ building blocks to generate distinct dihedral, octahedral and icosahedral assemblies on mixing (with *C*_3_-36B). Following the equimolar mixing of *C*_3_-36B with each of the new designed building blocks (individually), we observed ordered assemblies by nsEM that match the corresponding design models (Fig. 3b; dimer partner no. 1 generates D32-12, dimer partner no. 2 generates O32-17, dimer partner no. 3 generates I32-2, tetrameric partner no. 4 generates O43-14 and tetrameric partner no. 5 generates O43-36). By applying this design procedure recursively, we generated line topologies in which new assemblies can be sequentially created, and each pair of adjacent nodes is complementary. For example, a trimeric building block was designed to assemble with tetrameric partner no. 5, forming a new octahedral assembly (O43-9; Fig. 3a). Finally, we generated the ring topology by designing a set of four cyclic building blocks (three with *C*_3_ and one with *C*_4_ symmetry), each with complementary interfaces, enabling their assembly with two other partners. This resulted in two tetrahedral (T33-182 and T33-14) and two octahedral (O43-5 and O43-12) cages. nsEM characterization again confirmed assembly to the target architecture in each case (Fig. 3b).

Once the structure of an assembly has been confirmed by nsEM, the structures of both building block components are also validated as their structures cannot differ greatly from the design model (otherwise, the assemblies would not properly form). In the stepwise design calculations described above, the design success rate for cases in which one of the components was previously validated was higher (~30%–50%) than cases in which both components were newly generated (~10%–20%). For the above stepwise design effort, we typically only needed to experimentally test five or fewer designs to obtain the correct assemblies.

## Construction of three-component cyclic and dihedral assemblies

We next sought to extend our modular design strategy to three-component systems. We experimented with incorporating two distinct interfaces rather than a single interaction surface as in the previous cases. In our atomic bonding analogy, this corresponds to using two types of bonds rather than a single type to build up more complex architectures.

**Fig. 4:**
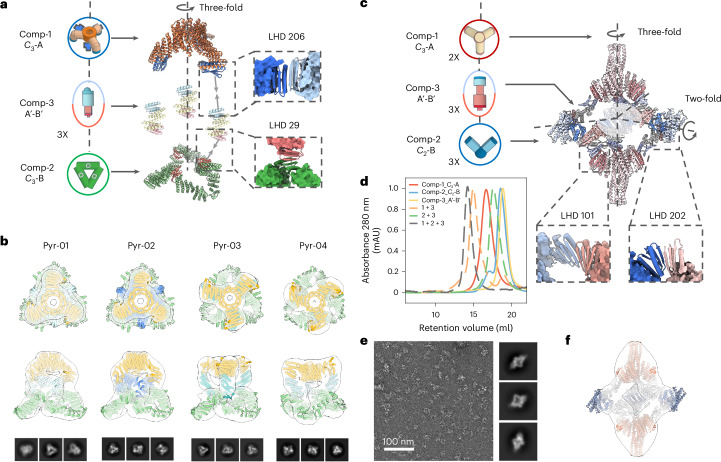
Design of three-component cyclic and dihedral assemblies using bispecific heterojunctions. **a**, Schematic of the co-axial connection of two cyclic *C*_3_ oligomers using designed bispecific heterojunctions. Protein backbones were generated by sampling along two degrees of freedom (translational and relative rotation between the two *C*_3_ oligomers). **b**, nsEM 3D reconstructions for four successful designs show good agreement with their design models. **c**, Heterojunctions can also be designed to generate dihedral assemblies by linking between prevalidated *C*_3_ and *C*_2_ cyclic oligomers. **d**, SEC elution profiles show that individual building blocks elute as monodisperse species (solid lines), and adding the heterojunction components to either the *C*_2_ or *C*_3_ building blocks does not trigger extended assemblies (green and orange dashed lines, respectively). The correct dihedral assemblies (grey dashed line) are the largest structures present when all three components are combined. **e**,**f**, Representative micrograph and 2D class averages (**e**) and 3D reconstruction (**f**) are close to the design model. Source data

We first used this approach to generate multicomponent cyclic oligomers. We began by attempting to generate higher-order *C*_3_-symmetric structures by connecting two *C*_3_ trimeric designs aligned but offset along their symmetry axes. We positioned different bonding interfaces on each of the stacked components of the *C*_3_ trimers, and kept one component fixed and sampled the rotations of the other around the symmetry axis and the translations along it. For each sampled placement, we designed rigid connectors with the two complementary bonding interfaces at each end with geometries crafted to exactly match the two bonding interfaces presented by the prepositioned *C*_3_ components. As illustrated in Fig. 4a, we used this approach to generate pyramid-shaped architectures with a narrow *C*_3_ cyclic oligomer at the apex, in which the monomers closely pack around the symmetry axis; a wider *C*_3_ ring at the base, in which the monomers surround a central cavity; and bispecific rigid connectors at the sides (based on LHD 206 and LHD 29 bonding modules, see the zoomed-in view). Out of the 24 designs that we experimentally tested, the nsEM 3D reconstruction of six designs revealed good matches to the corresponding design models (four out of the six are shown in Fig. 4b).

We next sought to use similar A′-B′ bispecific connectors to bridge *C*_3_ and *C*_2_ oligomers, forming dihedral assemblies. We used the *C*_3_ component *C*_3_-36B from the O43-36 cage (Fig. 3a), placed a *C*_2_ component with the symmetry axis intersecting the *C*_3_ axis at 90° and sampled the rotations of *C*_2_ around the *C*_3_ axis (Fig. 4c). We again designed bispecific connectors with two distinct bonding interfaces to bridge the corresponding interfaces on the *C*_2_ and *C*_3_ components. The SEC elution profiles show that all three components can be separately purified and have the intended oligomeric states as do the two assembly intermediates *C*_3_-A-connector and *C*_2_-B-connector (Fig. 4d). nsEM 2D class averages and 3D reconstructions of the full three component designed dihedral assembly are consistent with the design models (Fig. 4e,f).

## Design of dynamically reconfigurable 2D lattices

We further applied our bond-centric approach to generate reconfigurable 2D lattices. As small deviations from the desired structures can add up to considerable strain in unbounded structures, the design of these may require higher accuracy and rigidity than smaller closed structures. Perhaps because of this, previously designed 2D arrays have only been generated using the computational docking of natural cyclic oligomers with known crystal structures, and the success rates have been relatively low^15,16^ (~2%–5%).

**Fig. 5:**
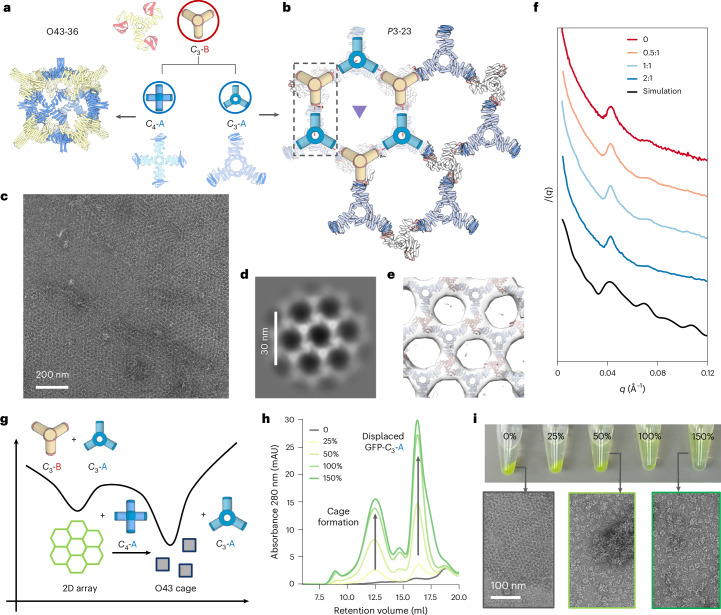
2D lattice design and reconfiguration dynamics. **a**, *C*_3_-B building block can be combined with *C*_4_-A to yield the previously characterized O43-36 cage, or assemble into a 2D array with *C*_3_-23A. **b**, *P*3-23 2D array unit cell with three-fold axes represented by a triangle. We initialized the lattice design by prepositioning one validated *C*_3_-36B oligomer (red) at the lattice sites, and set out to create the second *C*_3_ building block with constrained degrees of freedom (blue). **c**, Representative nsEM micrograph showing a micrometre-scale crystalline domain. **d**, Zoomed-in view of 2D class average (inset). **e**, Design model fitted in the reconstructed nsEM 3D map, showing good agreement. **f**, SAXS patterns for samples prepared at increasing equivalents of monodentate modulators (top to bottom), compared with the simulation results of a finite-sized 2D array. **g**, Schematic of the relative stability of cage versus layer assembly. **h**, SEC elution profiles and images (post-centrifugation) showing the dissolution of preassembled 2D protein arrays through dynamic exchange between the *C*_4_-36A and *C*_3_-23A proteins. As the *C*_4_-36A concentration increases from 0% to 150% (relative to *C*_3_-23A), higher levels of cages and *C*_3_-23A-GFP are detected in the mixture supernatant. **i**, nsEM micrographs showing transition from layer (left) to cage (right) on the addition of *C*_4_-36A proteins to preassembled 2D arrays during incubation at room temperature.

We explored whether the robust generative design of 2D arrays could be achieved using our modular bonding approach. Initially, we attempted the WORMS protocol and selected 24 two-component 2D layer designs for experimental validation, but only observed disordered aggregates. We attributed this failure to the extended structures generated by the WORMS’ additive fusion strategy, and turned to focusing on making more compact designs using RFdiffusion. We used a cyclic homotrimer *C*_3_-36B from the O43-36 cage as one of the two components (Fig. 3a). We placed a second component, *C*_3_-36B, to generate the plane symmetry group *P*3. With the *C*_3_-A design model fixed at the origin, we sampled the lattice spacing, the *z* offset of the *C*_3_-36B trimer from the lattice plane and the rotation of the trimer along its three-fold axis. We explicitly modelled only the asymmetric subunit (single chains from each oligomer) required to generate the full assembly (Fig. 5b). Three out of the six experimentally characterized *C*_3_-A components had SEC elution peaks consistent with the designed homotrimers. We next sought to assemble the lattice by combining equimolar *C*_3_-36B assemblies with *C*_3_-A, and observed immediate precipitation. Characterization of the precipitated material by nsEM revealed 2D arrays for two of the three designs. To improve long-range periodicity, we modulated the in vitro layer nucleation and growth dynamics by including a third component, GFP-labelled monodentate LHD 101A′, to compete with *C*_3_-36B binding to *C*_3_-A. The A′ ligands at concentrations ranging from 0.5 to 3 equivalents were added to temporarily cap *C*_3_-A; although these capping interactions form quickly, we anticipated that they would eventually be replaced by the more avid *C*_3_-36B trivalent components to form lattices (Fig. 5c). The nsEM 2D class averages confirmed lattice formation with the intended symmetry and lattice spacing (Fig. 5d), and a 3D reconstruction was closely superimposable on the design model (Fig. 5e). The addition of capping units greatly slowed aggregation for all the samples. We incubated the three-component mixtures at 50 °C overnight to facilitate *C*_3_-36B linker exchange to reach equilibrium. Small-angle X-ray scattering (SAXS) experiments suggested larger crystalline domain sizes with an increasing concentration of modulators (Fig. 5f).

Our building of multiple distinct architectures from combinations of a single common component with different architecture-specific components enables the exploration of the dynamics of assembly reconfiguration. The 2D array and O43-36 cage share the *C*_3_-36B building block, and we explored whether they can dynamically reconfigure. We first mixed equimolar of *C*_4_-36A (cage) and *C*_3_-23A (layer) and combined them with the *C*_3_-36B component. No obvious aggregates were observed, and nsEM revealed the formation of the O43-36 cage as the dominant species (Supplementary Fig. 4). This suggested that the formation of the bounded cage architecture is kinetically favoured compared with the unbounded 2D layer (Fig. 5g). Next, we performed a titration experiment by adding an increasing amount of *C*_4_-36A proteins to preassembled 2D arrays, where *C*_3_-23A was labelled with GFP and the assemblies formed green precipitates. At room temperature, we immediately observed array dissolution and cage formation, as evidenced by SEC elution profiles of the supernatant (Fig. 5h,i). Despite the assemblies being driven by the identical molecular binding interface, these results suggest that the O43-36 cage is thermodynamically more stable than the 2D layer, likely because of the formation of additional interactions upon assembly closure, and the kinetic barrier between the two assembly states can be overcome through dynamic exchange at room temperature.

## Hierarchical 3D assemblies with polyhedral building units

High-valency polyhedra are ideal building blocks for crystal engineering, facilitating network topologies unachievable with homo-oligomers and stabilizing highly porous structures. For example, preassembled metal–oxo clusters serve as secondary building units in the design of a variety of metal–organic frameworks^26^. We sought to design polyhedral cages that display outward-facing bonding modules that can act as secondary building units for protein crystal engineering.

**Fig. 6:**
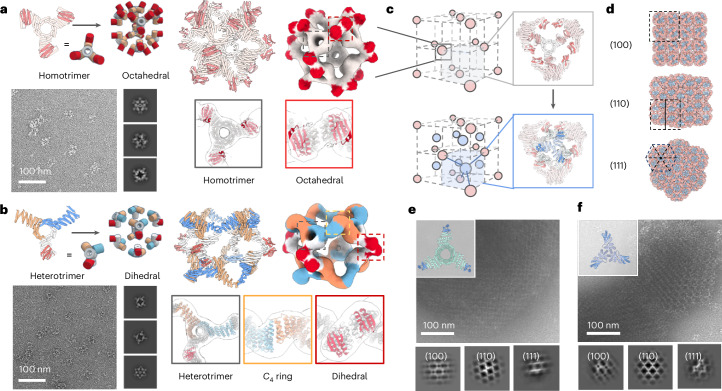
Design of high-coordination bonding units for 3D crystals. **a**,**b**, nsEM structural characterization confirms octahedral *O*_3_ (**a**) and dihedral *D*_4_ (**b**) assemblies with the available bonding modules from docked homotrimeric and heterotrimeric building blocks, respectively. **c**, Placing octahedral assemblies in a face-centred-cubic lattice arrangement exposes unsaturated bonding modules (top). The designed trimeric crystal linkers induce macroscopic crystallization by bridging between nearby octahedral cages to form a local T33 tetrahedral complex (bottom). **d**, View of the designed crystal (2 × 2 × 2 unit cells) along different directions. **e**,**f**, On mixing with the *O*_3_ cage, two different de novo trimeric linker designs *C*_3_-23 (**e**, inset) and *C*_3_-4 (**f**, inset) yielded crystalline assemblies, *O*_3__*C*_3_-*F*432-23 and *O*_3__*C*_3_-*F*432-4, respectively. The representative nsEM micrographs of the polycrystalline assembly and 2D class averages show good agreement with the designed crystals viewed along all the three major zone axes.

Octahedral assemblies are appropriate building blocks for 3D cubic lattices. We hence sought to design octahedral assemblies displaying 24 bonding modules. We docked eight designed *C*_3_ homo-oligomers into an octahedron using the RPXDock protocol^27^ such that LHD 206 is available for bonding (Fig. 6a, bonding modules highlighted in red). Experimental characterization confirmed the successful formation of *O*_3_ cages, with the nsEM 3D reconstruction closely matching the design model at both homotrimeric and interface regions (Fig. 6a).

**Extended Data Fig. 2:**
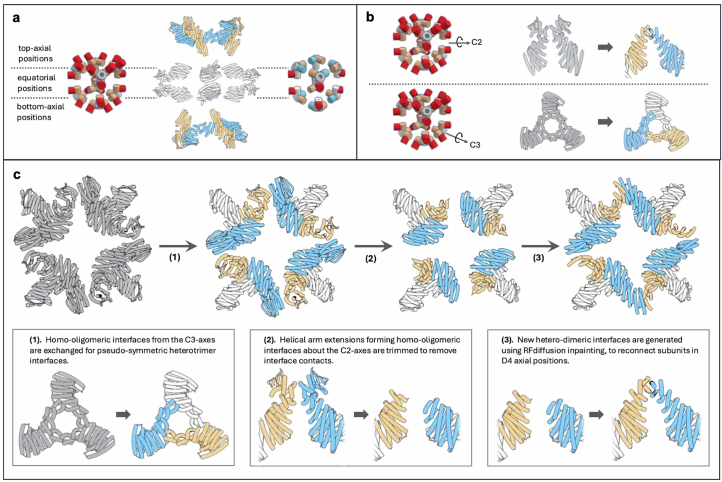
Conversion from O3 symmetry to D4 symmetry. **a**, breaking of symmetry about octahedral edges was performed by converting the homo-dimeric interfaces between 16 axial subunits to heterodimers, while the homo-dimeric interfaces between 8 equatorial subunits maintained their original C2 symmetry. **b**, interfaces about all C3 symmetry axes were exchanged for a geometrically analogous heterotrimer^1^, and interfaces about the axial C2 symmetry axes were exchanged for newly generated heterodimeric interfaces. **c**, exchange of axial homo-dimeric interfaces was performed using RFdiffusion and a 3-step protocol.

*D*_4_ symmetric assemblies are appropriate building blocks for 2D and 3D tetragonal lattices. We hence sought to design *D*_4_ dihedral cages with eight available bonding modules. To do this, we broke the perfect symmetry of the *C*_3_ homo-oligomer using a heterotrimeric variant^28^ containing only one bonding module (Fig. 6b and Extended Data Fig. 2). RPXDock was then used to create anisotropic dihedral protein assemblies with *D*_4_ symmetry. The nsEM 3D reconstruction again closely matched the design model (Fig. 6b, bonding modules highlighted in red).

We next designed 3D crystals in the *F*432 face-centred-cubic space group by assembling *O*_3_ cage secondary building units with complementary crystal linkers. As a test case, we designed a high symmetry cubic lattice in which four *O*_3_ cages in close proximity are interconnected by four designed *C*_3_ linkers to form a local tetrahedron (Fig. 6c). In this architecture, the *O*_3_ cages have only one translational degree of freedom along their diagonal *C*_3_ rotational axes. We generated rigid *C*_3_ linkers bridging the *O*_3_ cages with the complementary bonding module LHD 206 using RFdiffusion, and selected 24 designs for experimental characterization. In vitro crystallization experiments were performed by mixing equimolar amounts of purified *O*_3_ cages and designed *C*_3_ linkers at 5-µM monomer concentrations. White flocculation was observed for five samples, which nsEM showed to be polycrystalline assemblies (Supplementary Fig. 5). nsEM 2D class averages show good agreement with the design model view along all the three major zone axes and expected lattice spacing: [100], [110] and [111] (Fig. 6d–f and Supplementary Fig. 6). The observed small-crystalline domain size probably reflects the strong bonding module interactions, which could be weakened by introducing interface mutations, monodentate LHD capping ligands or excipients that modulate the crystallization kinetics.

## Discussion

The ability to design directional protein-bonding modules and use them to create highly precise bounded and unbounded nanomaterials through self-assembly represents an important advance in protein design. Key to this success is the programming of well-defined directional interactions, achieved by combining reversible heterodimeric interfaces with generative protein design to control their precise orientation. These standardized interfaces and bonding geometries are highly predictable, allowing the generation of a wide variety of scalable assemblies emerging from a small set of reusable building blocks, which greatly simplifies the design process, especially for 2D and 3D open structures. Our approach has the advantage over traditional fusion-based nanomaterial design methods of using standardized reconfigurable interfaces^20^ and custom-designed building blocks, which increases both programmability and success rates. Our approach also extends previous efforts to develop extendable platforms using single-component standardized protein blocks to multicomponent systems^19^. Leveraging deep learning-based generative protein design allows us to independently control the bonding geometry and interaction strength, ranging from dissociation constant *K*_d_ = 10 nM (LHD 101) to *K*_d_ = 2 µM (LHD 202)^20^, increasing the structural space that can be explored compared with atomic systems that are constrained by quantum mechanical principles. This capability could enable the exploration of anisotropic structures and quasi-symmetric phases with broken symmetry^29,30^, which are challenging to address with traditional methods. As shown in ref. ^31^, a similar strategy has been used to finely tune the size and shape of a series of bifaceted protein nanoparticles that can colocalize distinct biological entities.

Our re-use of the building blocks enables the rapid generation of new architectures from the substructures of previously validated assemblies, with increased success rates for cage designs that use such blocks. The ability of a single component to form multiple distinct assemblies, as highlighted by the *C*_3_ component (Fig. 3) that can be driven into five different nanocage assemblies depending on the added partner, provides not only an economy of coding but also opens up opportunities for storing information^32^ as the assemblies populated will depend on the order of addition. Our protein assembly networks could also be useful as logic gates^33^ that produce distinct outputs based on various inputs.

Our findings highlight the potential of computational protein design for developing designer nanomaterials, with a modularity approaching the capabilities of DNA nanotechnology^34^. Since the designed proteins are expressible in diverse living systems through genetic encoding, they hold promise for direct integration as structural, signalling and control units within living cells, opening new opportunities for cellular computing^35^. Just as standardized parts transformed industrial manufacturing, standardized protein subunits, which assemble according to simple rules, should facilitate the creation of protein assemblies for a wide range of applications.

## Methods

### Computational design strategy

#### Assembly backbone design with RFdiffusion

We used RFdiffusion to design symmetric homo-oligomers that rigidly hold bonding motifs such that they exactly match the presentation orientation of existing binding partners (Extended Data Fig. 1). In a typical input preparation, we first create a virtual building block *C*_3_-AB′, by symmetrically arranging fragments of the complementary binding partners for an existing cyclic oligomer *C*_3_-A. The outward-facing virtual building block *C*_3_-AB′ has a central cavity, but contains geometric constraints. Next, we symmetrically arrange *C*_3_ assemblies to sample rotations and translations along their new symmetry axes. New oligomeric binding partners were then isolated and created through symmetric RFdiffusion. For constructs generated using WORMS (for example, for pyramidal symmetry), these spatial configurations between the two *C*_3_ complexes were checked to see whether rigid fusions could connect the top and bottom subunits via a simple helix alignment. For constructs generated using RFdiffusion (dihedral symmetry), symmetric denoising was performed, to connect the top and bottom subunits with new *C*_2_-symmetric interfaces.

#### Backbone generation with WORMS

A library of cyclic oligomer scaffolds (*C*_2_, *C*_3_ and *C*_4_) from crystal structures deposited in the Protein Data Bank^18,36–39^ (http://www.rcsb.org/pdb/) and from previous de novo designs were used as the input scaffolds. To enable the generation of a diverse range of architectures, we guide the WORMS software with a configuration file to truncate inputs from the structural database and exhaustively for fusible bridging elements. The default WORMS settings were used, except that the ‘tolerance’ parameter was set to 0.1 from 0.25 to reduce closing error (‘tolerance’ defines the permitted deviation of the final segment from its targeted position within the structure). The number of backbone fusion outputs produced depends on the allowed fusion points and tolerance parameter, as the design space expands exponentially with the number of segments being fused.

#### Sequence design with ProteinMPNN

We performed three cycles of ProteinMPNN^40^ and Rosetta^41^ FastRelax to design sequences for backbones generated from RFdiffusion or WORMS protocol. For homomeric oligomer designs, it is possible to restrict the sequences to be identical between the structural elements where that is desired, using the –tied_positions argument as described.

#### In silico filtering

AlphaFold2 was used to assess whether our designed sequences will fold or assemble as intended. We primarily used the prediction results from Model 4 as it usually provided the highest-confidence predictions for all α-helical proteins. The computational metrics^23^ filtering cut-offs were set to predicted local-distance difference test (pLDDT) score > 90, predicted template modelling (pTM score) > 0.80 and Cα root mean square deviation of less than 1.5 Å or 2.0 Å compared with the ideal design model.

#### RPXDock cage docking and design

Homotrimeric and heterotrimeric rings were computationally docked to create backbone configuration for the *O*_3_ and *D*_4_ cages, respectively. The *O*_3_-symmetric cage was adapted to a three-component *D*_4_-symmetric assembly using RFdiffusion and interface exchange (Extended Data Fig. 2). The sequence of cage-contacting interfaces were redesigned by ProteinMPNN, following a rigorous Rosetta filtering^41^ process based on several metrics, including a methionine count of ≤5, shape complementarity of >0.6, change in Gibbs free energy (ddG) of less than –20 kcal mol^−1^, solvent-accessible surface area of <1,600, clash check of ≤2 and unsatisfied hydrogen bonds of ≤2. To improve the cage yield and reduce aggregation propensity, we further optimized their sequences using ProteinMPNN and filtered designs based on the change in spatial aggregation propensity (SAP) score of <30.

### Protein expression and purification

Synthetic genes from computationally filtered designs were acquired from IDT and cloned into the pET29b+ vector using NdeI and XhoI restriction sites. These designs were expressed in BL21* (DE3) *E. coli*-competent cells using a bicistronic system with a C-terminal polyhistidine tag. For protein expression, transformants were cultured in 50-ml Terrific Broth supplemented with 200 mg l^−1^ kanamycin and induced for 24 h at 37 °C under a T7 promoter. Cells were harvested by centrifugation, resuspended in Tris-buffered saline and lysed with 5 min of sonication. The lysates were then subjected to nickel affinity chromatography, washed with ten-column volumes of 40-mM imidazole and 500-mM NaCl, and eluted with 400-mM imidazole and 75-mM NaCl. Successful complex formation was confirmed by the presence of both oligomers on sodium dodecyl sulfate–polyacrylamide gel electrophoresis following Ni-NTA pulldown. Proteins of the correct molecular weights were further analysed by electron microscopy. Selected designs were scaled up to 0.5 l for additional expression and purification under the same conditions. The in vitro assembly of complexes was achieved by mixing individually purified components at equimolar ratios, with 18 assemblies displaying SEC profiles consistent with the designed oligomeric states.

### nsEM

Cage fractions obtained from the SEC traces or by in vitro mixing were diluted to a concentration of 0.5 µM (monomer component) for characterization by nsEM. A 6-µl sample of each fraction was placed on glow-discharged, formvar/carbon-supported 400-mesh copper grids (Ted Pella) and allowed to adsorb for over 2 min. Each grid was blotted and stained with 6 µl of 2% uranyl formate, blotted again and restrained with an additional 6 µl of uranyl formate for 20 s before the final blotting step. Imaging was performed using a Talos L120C transmission electron microscope operating at 120 kV.

All the nsEM datasets were processed using CryoSparc software. Micrographs were uploaded to the CryoSparc web server, and the contrast transfer function was corrected. Approximately 200 particles were manually selected and subjected to 2D classification. Selected classes from this initial classification served as templates for automated particle picking across all the micrographs. Subsequently, the particles were classified into 50 classes through 20 iterations of 2D classification. Particles from the selected classes were utilized to construct an ab initio model. Initial models were further refined using *C*_1_ symmetry and the corresponding *T*/*O*-symmetry adjustments.

### Cryo-EM sample preparation, data collection and processing

#### T33-549 cage

T33-549 solution (8.5 mg ml^−1^ in 25 mM of Tris (pH 8) with 300 mM of NaCl) was diluted 1:9 in a sample buffer, and then, the grids were immediately prepared using Vitrobot Mark IV in which the chamber was maintained at 22 °C and 100% humidity. Then, 3.5 µl of diluted T33-549 (final concentration, ~0.9 mg ml^−1^) was applied to the glow-discharged surface of grids (QUANTIFOIL R 2/2 on Cu 300 mesh + 2-nm C) and then immediately plunged into liquid ethane after blotting for 4 s with a blot force of 0. Grids were first screened at the NYU Cryo-Electron Microscopy Laboratory on a Talos Arctica microscope operated at 200 kV and equipped with an energy filter and Gatan K3 camera. Data were then collected at the National Center for Cryo-EM Access and Training (NCCAT) at the New York Structural Biology Center on a Titan Krios microscope operated at 300 kV with a Gatan K3 camera. Furthermore, 12,276 videos were collected, and all data acquisition was controlled using Leginon^42^. The data acquisition parameters are shown in Supplementary Table 4.

The data processing workflow is described in Supplementary Fig. 2. Videos were imported into CryoSPARC^43^ for processing and split into 13 subsets during the initial processing steps. After patch motion correction and contrast transfer function estimation, images were curated, leading to the removal of 696 micrographs. Another 253 micrographs were randomly selected to generate templates using both manual picking and blob picker, and the picked particles were fed into 2D classification jobs. The resulting templates (14,616 particles from the 5 best classes) were used to train Topaz (conv127)^44^, which was then used to pick all micrographs. The resulting 4,841,024 particles were extracted at 4.94 Å pixel^−1^ and two rounds of 2D classification were carried out, followed by the removal of duplicate particles for each of the 13 subsets of micrographs. The resulting 1,058,870 particles were then grouped into three subsets for further processing. One of these groups was used to generate an ab initio model (using *T* symmetry). Each of the three subsets was then fed into 3D homogeneous refinement jobs, leading to ~10-Å models. After 3D heterogeneous refinement in *C*_1_ symmetry, bad classes were removed, leading to 751,758 particles among the three subsets. Particles were re-extracted at 1.24 Å pixel^−1^ before another round of 3D refinement without symmetry applied and another round of 3D heterogeneous refinement. The best classes of the three subsets were then merged, leading to an ~7.0-Å resolution map (*C*_1_), and two more rounds of non-uniform refinements^45^ were performed, leading to a resolution of 6.8 Å without symmetry (*C*_1_) and 6.1 Å with *T* symmetry (Supplementary Fig. 3a,b). The map using *T* symmetry has a sphericity^46^ of 0.972 (unmasked). The design model was then docked as a rigid body into the resulting map using Chimera UCSF^47^ (Supplementary Fig. 3c), followed by conservative real-space refinement in Phenix^48^ (Supplementary Fig. 3d), with constraints on the secondary structure (Supplementary Table 5).

#### O42-24 cage

2 µl of the cages at a concentration of 1.3 mg ml^−1^ in 150 mM of NaCl and 25 mM of Tris (pH 8.0) was applied to glow-discharged QUANTIFOIL R 2/2 on Cu 300-mesh grids + 2-nm C grids. The grids were plunge frozen in liquid ethane using Vitrobot Mark IV, with a wait time of 7.5 s, blot time of 0.5 s and a blot force of –1. A total of 3,196 videos were collected in the counting mode, each consisting of 75 frames, using a Titan Krios microscope operating at 300 kV and equipped with an energy filter. The pixel size was 0.84 Å, with a total dose of 61 *e*⁻ Å^−^^2^ per video.

All data processing was carried out using CryoSPARC v. 3.3.2 (ref. ^43^). Patch motion correction and patch contrast transfer function estimation were performed using default parameters. An initial set of 224,225 particles was picked using the blob picker tool, followed by extraction at a box size of 640 pixels and Fourier cropping to 320 pixels. 2D class averages were generated, and the nine best classes were low-pass filtered to 20 Å to serve as references for template-based particle picking, resulting in a refined set of 185,832 particles. These particles were re-extracted using a 640-pixel box size and Fourier cropped to 320 pixels. A subsequent 2D classification into 100 classes identified 69,634 high-quality particles, which were used for ab initio 3D reconstructions, sorted into three classes with octahedral symmetry applied. Non-uniform refinement, using the best ab initio map as the initial model and all 69,634 of the best particles from the 2D classification, yielded a final 3D map with a global resolution estimate of 8.3 Å.

### SAXS data collection and pattern simulation

SAXS was performed on a Xenocs Xeuss 3.0 instrument with an X-ray energy of 8.04 keV (wavelength, 1.54 Å) using a Cu Kα microfocus source. Data were collected in three configurations: low-*q* (0.003–0.007 Å^−1^) for 18,000 s, mid-*q* (0.007–0.020 Å^−1^) for 10,000 s and high-*q* (0.020–0.200 Å^−1^) for 7,200 s. Samples were loaded in 1.5-mm-diameter thin-walled quartz capillary that were purchased from Charles Supper. Data reduction was performed by subtracting the background from another capillary with the water solvent. Data reduction and merging were performed using the XSCAT software (v2.10.3).

The simulated small-angle scattering curves of the computational models of the protein crystals were calculated by using a Monte Carlo sampling of the Debye equation. This method allows for a fast and accurate calculation of the scattering curve of large structures^49,50^. In short, the atomic coordinates of each atom were first extracted from the Protein Data Bank file of the protein crystal. X-ray scattering length densities^51^ were then assigned to each atom. Two random coordinates were then selected and the distance between these points was calculated. After sampling 10 million pairs of random coordinates, the pairwise distribution was created, which was then transformed into the scattering curve using Fourier inversion. The code and notebook used to perform this simulation is available online (https://github.com/pozzo-research-group/MC-DFM/tree/main/Notebooks).

## Online content

Any methods, additional references, Nature Portfolio reporting summaries, source data, extended data, supplementary information, acknowledgements, peer review information; details of author contributions and competing interests; and statements of data and code availability are available at 10.1038/s41563-025-02297-5.

## Supplementary information

Supplementary Information Supplementary Figs. 1–6 and Tables 1–5.

## Source data

Source Data Fig. 1 Combined SEC source data for D32-6, T33-158, O43-60 and O43-68.

Source Data Fig. 2 SEC source data.

Source Data Fig. 3 SAXS pattern source data.

Source Data Fig. 4 SEC source data.

## Data Availability

All data are available in the Article or Supplementary Information. Structural coordinates of the assembly design model examples are available via Zenodo at 10.5281/zenodo.14537926 (ref. ^52^). Documentation for RFdiffusion^11^, ProteinMPNN^40^ and RPXDock^27^ is available via GitHub at https://github.com/RosettaCommons/RFdiffusion, https://github.com/dauparas/ProteinMPNN and https://github.com/willsheffler/rpxdock, respectively. Designs filtering with AlphaFold2 (ref. ^23^) is available via GitHub at https://github.com/google-deepmind/alphafold (Methods). Example commands for symmetric RFdiffusion motif scaffolding and automated sequence design pipeline of assemblies are available from the corresponding authors on request. For the T33-549 cage structure, the coordinates are deposited in the Protein Data Bank with accession code 9DRL and the cryo-EM density maps are deposited in the Electron Microscopy Data Bank with accession code EMD-47128. Source data are provided with this paper.
