# Supplementary material for: Bond-centric modular design of protein assemblies
Source: Nat Mater. 2025 Jul 31;24(10):1644–52. doi: 10.1038/s41563-025-02297-5 (PMC12484077; doi:10.1038/s41563-025-02297-5)
Supplement: Supplementary file 1 — Supplementary Figs. 1–6 and Tables 1–5. [file 41563_2025_2297_MOESM1_ESM.pdf]

nature materials

Supplementary information https://doi.org/10.1038/s41563-025-02297-5

Bond-centric modular design of protein assemblies

In the format provided by the authors and unedited

Table of Contents

Supplementary Figures 1-6

Supplementary Table 1-5

Supplementary References

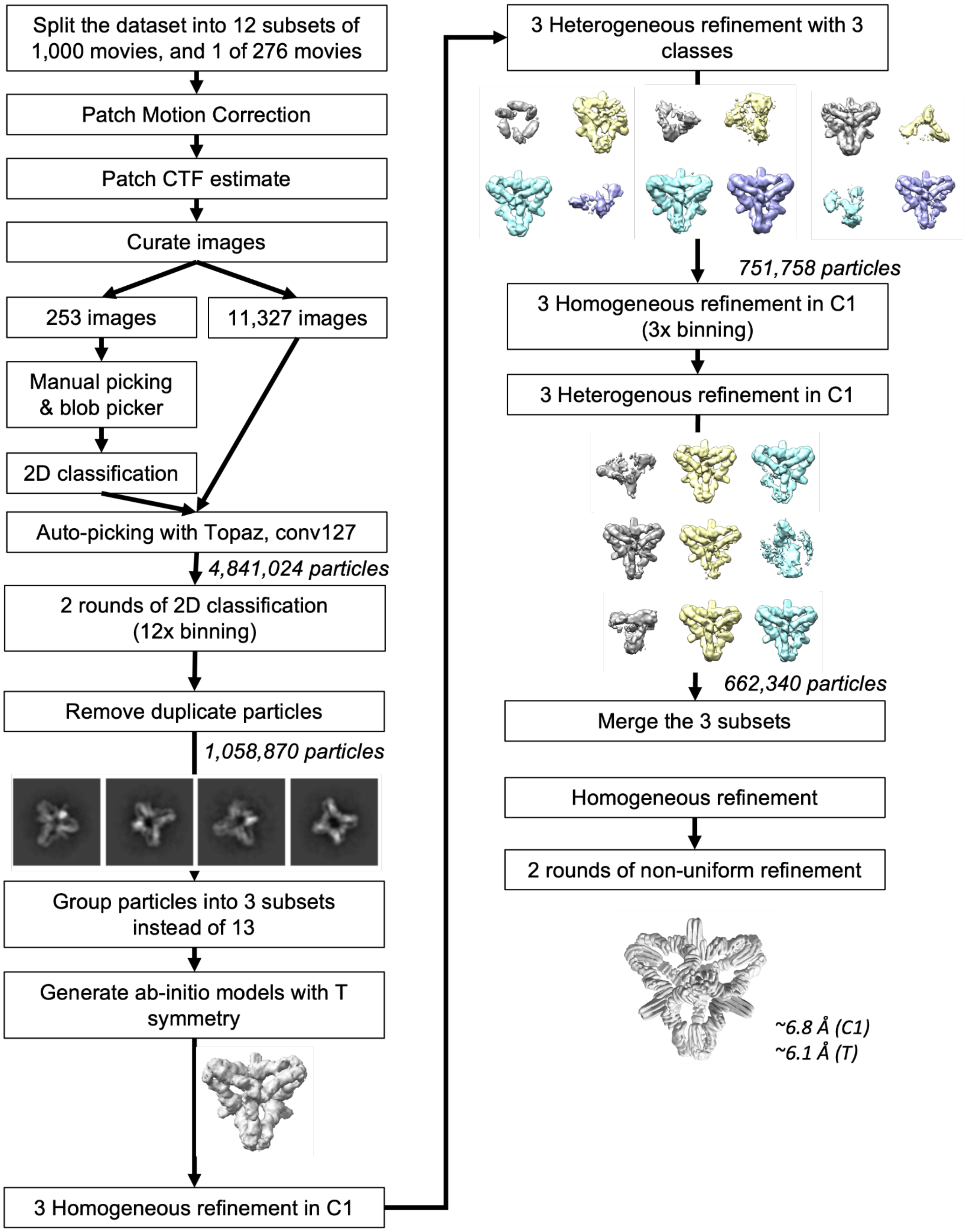

Fig. S1. Data processing workflow for T33-549 cage.

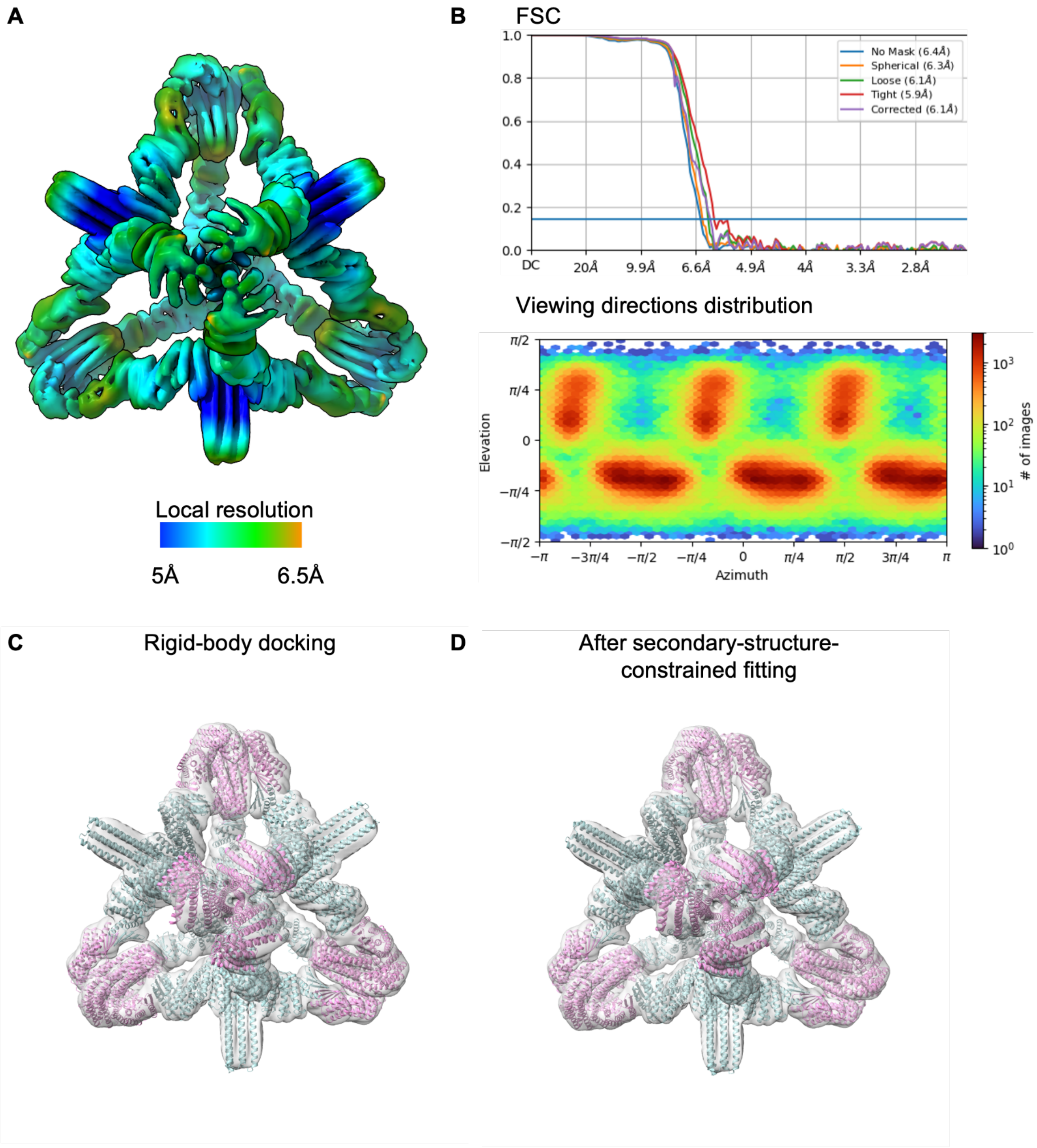

Fig. S2. Cryo-EM maps of the T33-549 cage. (A) Local resolution as estimated by CryoSPARC. (B) Overall resolution and viewing direction distribution from CryoSPARC. (C) Original designed model rigid-body docked into the cryo-EM map. (D) Same model after refinement in Phenix with secondary-structure constraints.

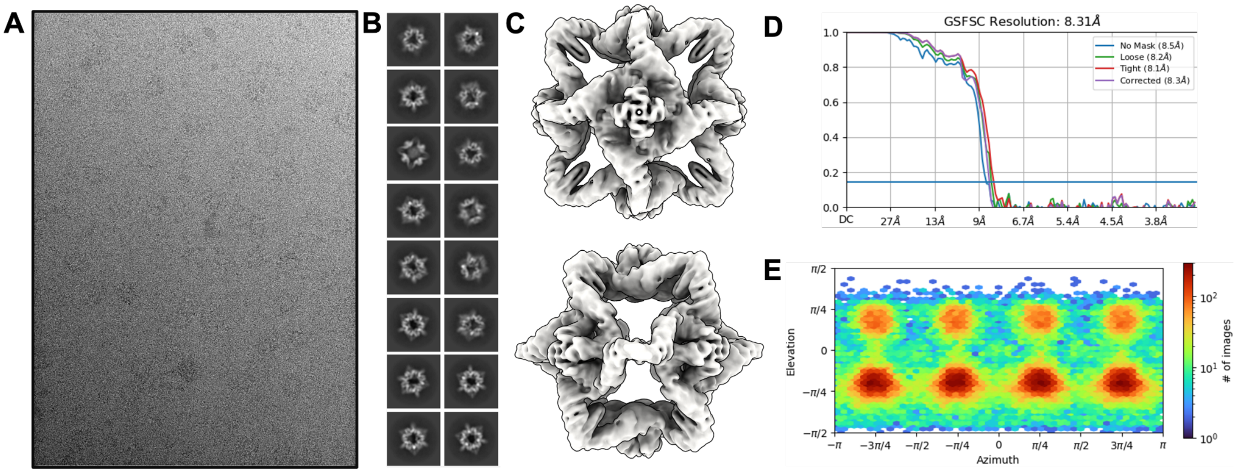

Fig. S3. Cryo-EM Data Collection and Processing of O42-24. (A) Representative raw micrograph illustrating the quality of collected data. (B) Representative 2D class averages of O42-24 particles, demonstrating distinct structural features. (C) 3D reconstruction of O42-24 at 8.31 Å resolution, with octahedral symmetry imposed. (D) Global resolution estimation using the Fourier shell correlation (FSC) cut-off value of 0.143. (E) Angular distribution plot showing uniform sampling of particle orientations across different views.

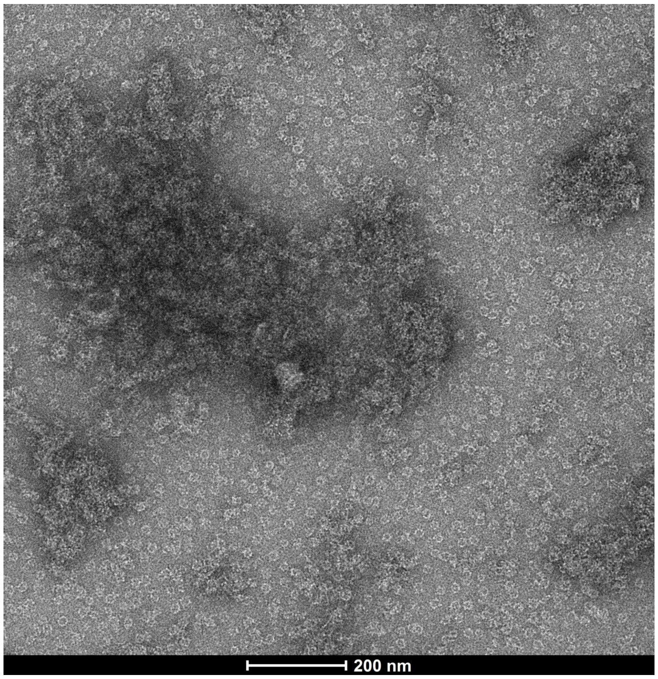

Fig.S4. A representative nsEM micrograph shows that the dominating assembly formed was the O43-36 cage and random aggregates, when C4-36A (cage component) and C3-23A (layer component) were mixed in a 1:1 molar ratio and combined with the C3-36B component *in vitro*.

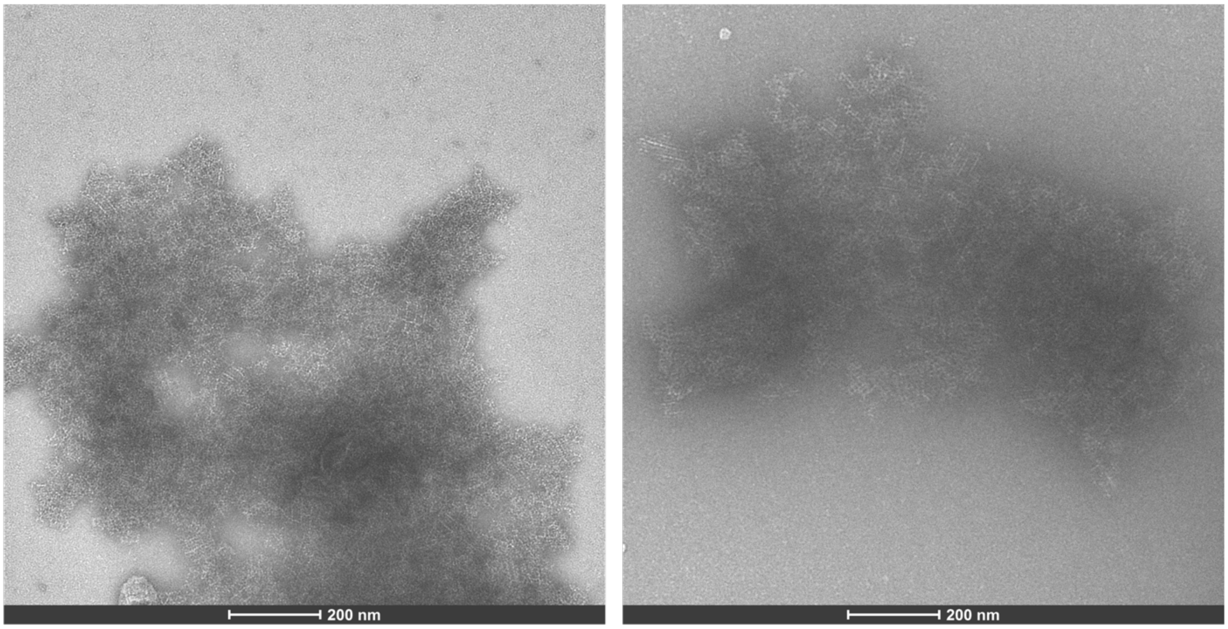

Fig.S5. Representative nsEM micrographs demonstrating that *in vitro* mixing of the O3 cage with designed trimeric linkers (unsuccessful designs) often yields polycrystalline domains, a common failure mode.

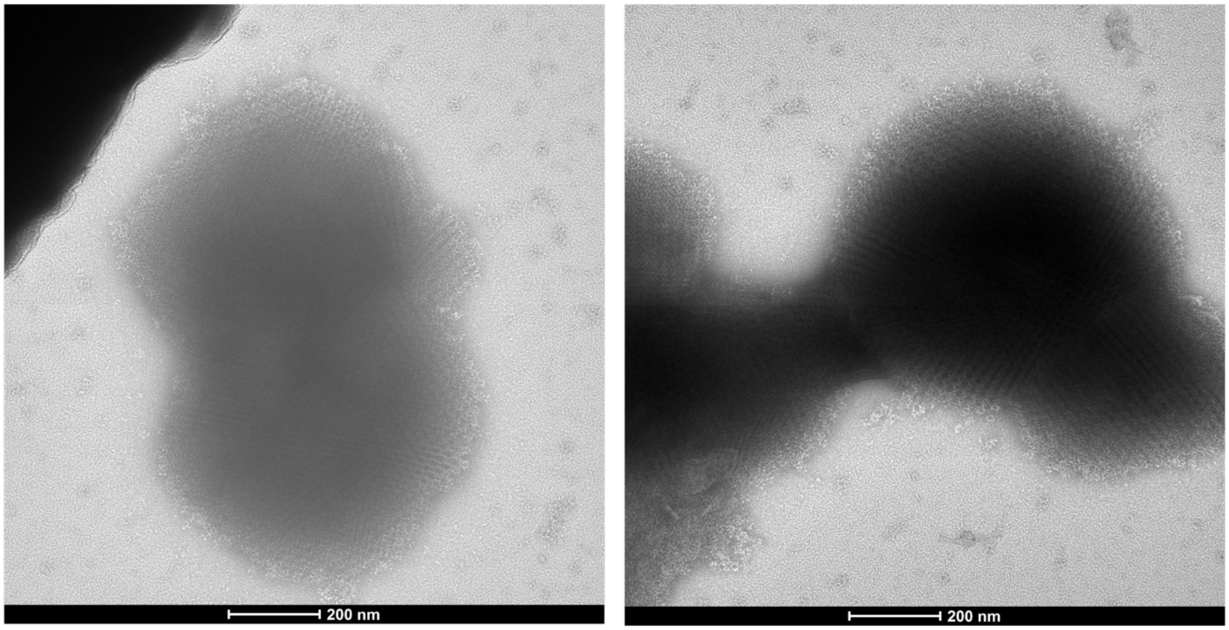

Fig.S6. Additional nsEM micrographs of *in vitro* assembled O3_C3-*F*432-23 design forming sub-micrometer crystalline domains.

Supplementary Table 1. Intersecting angles between symmetry axes used for different cage architectures.

T32	T33	O32	O42	O43	I32	I52	I53
C3-C2	C3-C3	C3-C2	C4-C2	C4-C3	C3-C2	C5-C2	C5-C3
54.7	70.5	35.3	45.0	54.7	20.9	31.7	37.4

Supplementary Table 2. Unique symmetry elements for crystals.

**P3**											
Symmetry Element	nFold	type	X	Y	Z	coordinate	X	Y	Z	Screw axis	
C3	3	AXS	0	0	1	CEN	0	0	0		
C3	3	AXS	0	0	1	CEN	-0.3333	0.3333	0		
C3	3	AXS	0	0	1	CEN	0.3333	0.6667	0		
C11	1	AXS	0	0	1	CEN	0	0	0	HEL	1
**P432**											
C2	2	AXS	1	1	0	CEN	0	0	0		
C2	2	AXS	1	0	0	CEN	0	0	0.5		
C2	2	AXS	1	1	0	CEN	0	0	0.5		
C3	3	AXS	1	1	1	CEN	0	0	0		
C4	4	AXS	0	0	1	CEN	0	0	0		
C4	4	AXS	0	0	1	CEN	0	-0.5	0.5		
C21	2	AXS	-1	1	0	CEN	0.5	0	0	HEL	0.7071
C21	2	AXS	-1	0	1	CEN	0.5	0.5	0	HEL	0.7071
C31	3	AXS	1	-1	1	CEN	0.3333	0.3333	0	HEL	0.5774
C32	3	AXS	1	1	-1	CEN	0.3333	0	0.3333	HEL	1.1547
O	O43	AXS	0	0	1	CEN	0	0	0	AXS2	001
O	O43	AXS	1	0	0	CEN	0.5	0.5	0.5	AXS2	100
D4	4	AXS	0	0	1	CEN	0	0	0.5	AXS2	001
D4	4	AXS	1	0	0	CEN	0	0.5	0.5	AXS2	100

Supplementary Table 3. Protein sequences experimentally tested in this study.
Note: *His-tag and Trp-tag are included in the listed sequences and can be removed. N-terminal methionines are omitted. Original design model PDBs are available on Zenodo: https://doi.org/10.5281/zenodo.14537927. Several designs were further optimized using ProteinMPNN to improve solubility; therefore, the sequences provided here may differ from those in the original design models. The recommended sequences used for experimental validation are shown below.

Name	Sequences*	Symmetry	Bonding motif
**2-component cage designs**			
O42-24_A	SALEKIAKLIIEAARASADVARRAARRAAEEARKAIED<br>VSTERGSSSLLKEVADRIVTMQEEVVRKIIIMQEIAAQ<br>LATILIKAAKEAASDESKMEDVAKTVQTLLTAAKLAMS<br>PDPEDLKEAVRLAEEVVRDKPGSEAANVALQIILLAA<br>EALAKLPDPEALKEAVKAAEKVVREQPGSNLAKKAL<br>EIILRAAAALANLPDPESRKEADKAADKVRREQPGSE<br>LAVVAAIISAVARMGVKMELHPSGNEVKVVIKGLHIKQ<br>QRQLYRDVREAAKKAGVEVEIEVEGDTVTIVVRGGS<br>WSGLEHHHHHH	C2	LHD101A
O42-24_B	ASSWVMLGLLLSLLNRLSLAAEAYKKAIELDPNDALA<br>WLLLGSVLLLLGREEEAEEAARKAIELKPEMDSARRL<br>EGIIELIRRAREAAERAQEAAERTGDPRVRELARELK<br>RLAQEAAEEVRRDPDSKDVNEALKLIVEAIEAAVRAL<br>EAAERTGDPEVRELARELVRLAVEAAELVQQDPSAS<br>TVSEALKIVVQMIDMAVEALEKAERTGDPKEREDARE<br>IVRMAVRAADIALRIAKVFKRIEKRPDDDEAVETAVRL<br>AREAKKVAERLQEMAKRKGDAKLLRAALLALEMAVR<br>AVELAIKSNPDNDEAVETAVRLARELAKVAEELIERAK<br>KTGDKELLKLAKRALEVAMRAVSLALKSNPDNEEAR<br>RVAAELVLLVIRAAVIEMGVTMEEHRSGNRVKVVIKG<br>LHESQQEQLLEDVLRAAEIAGVRVRIRFKGDTVTIVV<br>EGLEHHHHHH	C4	LHD101B
T33-549_A	GSEEKIEKLLEELTASTAELKRATASLRAITEELKKNP<br>SEDALVEHNRAIVEHNAIIVENNRIIATVLLAIVAAIATN<br>EATLAADKAKEAGASEVAKLAKKVLEEAEELAKEND<br>SEEALKVVKAIADAAKAAAEAAREGKTEVAKLALKVL<br>EEAIELAKENRSEEALDVVRAIALAAYAAARAAQAGA<br>TDFAKDALRKLEEAIEEAKKRRSEKALREVYTIALKAA<br>VQAAEAAVRAQPGSNRAKSALEIILQAAEELAKLPDP<br>EALKDAVKAAEKVVREQPGSNLAKKALEIILRAAAAL<br>ANLPDPESRKEADKAADKVRREQPGSELAVVAAIISA<br>VARMGVKMELHPSGNEVKVVIKGLHIKQQRQLYRDV<br>REAAKKAGVEVEIEVEGDTVTIVVRGLEHHHHHH	C3	LHD101A

T33-549_B	GGKELEIVARLQQLNIELARKLLEAVARLQELNIDLVRKTSELTDEKTIREEIRKVKEESKRIVEEAEQEIRKAEAESLRLTAEAAADAARKAALRMGDERVRRLAAELVRLAQEAAEEATRDPNSSDQNEALRLIILAILAAVKALDAAIRTGDPEVRELARELVRLAVEAAEEVQRNPSSSDVNEALKLIVEAIEAAVQALEAAIEAGDPREREKARELVRLAVEAAEEVQRNPSSKEVNVKLKAIVVAIKVFVLKLSGTSEDEIAEEIARDISELIRKLKEDGSSYEDICEAVATVVDMVVEALKRAGTSEDEIAEIVARVISEVIRTLKESGSSYEVICECVARIVAAIVEALKRSGTSEEEIAEIVARVIQEVIRTLKESGSSYEVIRECLRRILEEVIEALKRSGVDSSEIVLIIIKIAVAVMGVTMEEHRSGNEVKVVIKGLHESQQEELLELVLRAAELAGVRVRIRFKGDTVTIVVRGGGWGGSENLYFQSGGLEHHHHHH	C3<br>LHD101B
D32-6_A	SEKARIAVENLEAALRLNRAAIEMAKSAEKILRDNPSDEKAQRYFTLTMRVLLMSLRLLYASLRLAEKALREEGSDDSAEKVRKEAEKILAESAKEMVRVAEVAVRFSPGSEEAKEALEAILTAAALLANAPDPEARKEADKAADKVRREQPGSELAVVAAIISAVARMGVEMELHPSGNEVKVVIKGLHIKQQRQLYRDVREAAKKAGVEVEIEVEGDTVTIVVRGGSWSGLEHHHHHH	C2<br>LHD101A
D32-6_B	EEAELAYLLGELAYKLGEYRIAIRAYRIALKRDPNNAEAWYNLGNVYYKRGDYDEAIEYYQKALELDPNNAEAWYLLGQAYLDQGDLDEAIEYMQKALELDPNNAEAKLALGAAKAAQSAQEAAERTGDPRVRELARELTRLATEAAREVIRDPSSSDVNEALKLINEAIEAASRALEAAERAGIPKVREIARLLVEAAVRAAERVQRNPSSSKDNETLKEIVRAIEEAVRALERAIQEGDPAERLRAAARAAQLAAKITGDAEALKEALRLLEKAVEIVEKRIKERPDDDKAVEQAVRLARELAKVAEELIERAKKTGDKELLKLAKRALEVAMRAVSLALKSNPDNEEARRVAAELVLLVIRAAVIEMGVTMEEHRSGNRVKVVIKGLHESQQEQLLEDVLRAAEIAGVRVRIRFKGDTVTIVVEGLEHHHHHH	C3<br>LHD101B
T33-158_0A	EEAELAYLLGELAYKLGEYRIAIRAYRIALKRDPNNAEAWYNLGNVYYKNGDYDEAIEYYQKALELDPNNAEAWYNLGMAYAKNGDYEEAIKYFEKALELDPNNIYAKALLAAAKAAKAAKEAGNEEAAKIAKELAKRAEEAYENGDKERAELAKKLAEALKLAISEDPEALKKAAELAKKVVERAPGSEEAKLALKVILLAAKKLAESEDPEALKAAAEFAKKVVERAPGSELAKEALKVIAEAAAKLASSPDPEALKAAEEFAKKVIERAPGSVLAVVAAIISAVARMGVKMELHPSGNEVKVVIKGLHIKQQRQLYRDVREAAKKAGVEVEIEVEGDTVTIVVRGLEHHHHHH	C3<br>LHD101A

T33-158_15B	EEAELAYLLGELAYKLGEYRIAIRAYRIALKRDPNNAEAWYNLGNVYYKNGDYDEAIEYYQKALELDPNNAEAWTNLGNAYLNQGDYDNAIKYYNKALELDPNNLAAKAALAAAKAAKAAQEKGDKEAAKAAKLLAEKAIEAYENGDEEAARAALAAAKALLAALKALKKAQEKGDEEAAKKAKELIKKAKEAAEKLDEVEALKVAVEALLKRVKLKPEDEEAIEELVEAARELKEEAERLRRRAREEGDPELLRRAVEALKEGVKAVLKAVEAKPEDEEAIEELVELAKELKEAAEELIRLAREEGDPELLEEARRALKEGLKAVGKALEAKPEDEEARELAAELVLLVIRAAVIEMGVTMEEHRSGNRVKVVIKGLHESQQEQLLEDVLRAAEIAGVRVRIRFKGDTVTIVVEGLEHHHHHH	C3<br>LHD101B
O43-60_13A	GSELEIVIRLQILNLELARKLLEAVARLQELNIDLVRKTSELTDEKTIREEIRKVKEESKRIVKEAEDEIKKAALISADLAAKAIKRAIDRAKKLLEKGEKEDAAKVLEKAAKEIEEVWKKLAEIAKNASTAEEALKAAKVIAELIKQLIEIARLLKEAGEKEKAAKVLEKAAKWAKEVAKLLEEIAKNAETEEEAKKAKEVIKELIKQLVEIARLLKEAGEEEKAQKVLLEAAKRLAELEDPEAQKEAIELAEKVVEREPGSELAKKALEVILEAAKKLAELEDPEAQKEAIEAAEKVVEREPGSELAKKALEVILEAAAKLASLPDPEAQKQAIEAAEKVIKREPGSVLAVVAAIISAVARMGVKMELHPSGNEVKVVIKGLHIKQQRQLYRDVREAAKKAGVEVEIEVEGDTVTIVVRGLEHHHHHH	C3<br>LHD101A
O43-60_6B	ASSWVMLGLLLSLLNRLSLAAEAYKKAIELDPNDALAWLLLGSVLLLLGREEEAEEAARKAIELKPEMEEAKVLLEAVRLIREAREAAERARKAAKEAGDPELEKRAEELAEELRRTAAEVRADPANEEARRRLRLVIRRTEAFVRAIENNLRLKEDPEDREAREEAAETVVEAIRAAVEEFGVTMEEHRSGNRVKVVIKGLHESQQEQLLEDVLEIAEIAGVRVRIRFKGDTVTIVVEGLEHHHHHH	C4<br>LHD101B
O43-68_4A	EEAELAYLLGELAYKLGEYRIAIRAYRIALKRDPNNAEAWYQLGNANYSNGDYDEAIEYYQKALELDPNNAEAWYNLGAAYASNGDYDEAIKYLKKALELDPNNLYAKALLAAAEAGDKEGAKVAIKLAELAKVAKENKDEELLKLVEKLARALLLAASPDPEAQKKAIELAWKVVKEQPGSELAKLALKVILIAAKKLAQSPDPEAQKQAIEAAWKVVKEQPGSELAQKALQVIAEAAAKLASSPDPEAQKQAIEAAQKVIDEQPGSELAVVAAIISAVARMGVKMELHPSGNEVKVVIKGLHIKQQRQLYRDVREAAKKAGVEVEIEVEGDTVTIVVRGLEHHHHHH	C3<br>LHD101A
O43-68_7B	ASSWVMLGLLLSLLNRLSLAAEAYKKAIELDPNDALAWLLLGSVLLLLGREEEAEEAARKAIELKPEMDSARRLEGIIELIRRAREAAERAQEAAERTGDPRVRELARELKRLAQEAAEEVRRDPDSKDVNEALKLIVEAIEAAVRAL	C4

	EAAERTGDPEVRELARELVRLAVEAAEEVQRNPSNKDVNEALKLIVEAIEAAVRALEAAERTGDPELRERARELVKRAVETARKVLENPSNEEVRIELRVEVAEILKEVARVYMELARREGDPELLKQAVEDLKEALELCKEAIKEDPENEEAIEEAVEIAKELKRAAEELIELARREGDKELLKQAKEALKEGLEACREAIKADPENEEAREVAAELVLLVIRAAVIEMGVTMEEHRSGNRVKVVIKGLHESQQEQLLEDVLRAAEIAGVRVRIRFKGDTVTIVVEGLEHHHHHH	LHD101B
O42-18_6A	TTEDIIEVLKLSLEVQKELAEQLKQLLEKLEQLQRKGQSDEEVRELLEKIKRLVQLIEELAEVQRKLVEVLKLLQENDSKKIYELSLEVVEKLKELAKESGIEKLAKIAVKVLEALGLLLVKLGDVKKVKEVLEKIKELAKESGTEEAAKEALKAIARIGAEAGKLGNLKAVEVAAEAARELAKEMGTEEAKKEALKALLRIARALAESPDIKALKAAIELAWKVVKETPGSELAKEALWVICEAAARLASSPDPEARKAAIEAAGKVVKETPGSPLAVVAAIISAVARMGVKMELHPSGNEVKVVIKGLHIKQQRQLYRDVREAAKKAGVEVEIEVEGDTVTITVKELEHHHHHH	C2<br>LHD101A
O42-18_1B	ASSWVMLGLLLSLLNRLSLAAEAYIKALKLDPNAKDAAELFGEVAEKLKLEEVARAAKLLARALELIEKARKTGDPELLREALRELKEAIRLVEEALRKDPELRYAIELAVLLAKQLKKIAELLAEEARKTGNPELLREALEALKEAIRAVEEALRADPENEEAIELAVELAKKLAELAEEFIEEARKTGNRELLREAREALEEGLRAVREALRADPENEEARELAAELVLLVIRAAVIEMGVTMEEHRSGNRVKVVIKGLHESQQEQLLEDVLRAAEIAGVRVRIRFKGDTVTIVVEGLEHHHHHH	C4<br>LHD101B
	Partner cage designs	
O43-36_B (**shared component**)	SDEAIKRLLEELRASTAELKRATASLRAITEELKKNPSEDALVEHNRAIVEHNAIIVENNRIIAAVLEAIVAEFILAKARLLVWEARKKGNPEEVLEAAKKALRVAEEAAKAGIEAIFKAAAEAALEIAMRLIDLATKKGDPDLVREAQKVAERVAELADKNGDEEVKLKAYAAAVAAEIIRRIETLKRSGSSYEDIRQTLRETLERIIEELKRRGVDSSQIVWAIIYVAVAVMGVTMETHKSGNEVKVVIKGLHESQQEELLELVLRAAELAGVRVRIRFKGDTVTIVVRGLEHHHHHH	C3<br>LHD101B

D32-12	SALEKIAKLIIEAARLTAEIAREAARAAAELARRAIEAV<br>SRERGSSSLLKAVADRIVEAQEQVVRLIIRQQQVAAK<br>LAEDLIRAAKEAASDEEKMERVAKDVRARALAAAAE<br>AMTELARAAGDPELLRRALELLERAVEIVTEAIRENP<br>DNDRAVELAVELARALKKIAEELQERAKKTGDPELLK<br>LALEALEVAVEAVTLAIKSNPDNDEAVETAVRLARELL<br>KVAEELQERAKKTGDPRLLLEALEALAVGVAAVTLAI<br>KSNPDNEEARETARRLAEELEKVAELLEERAKETGD<br>PSLQAAARAARAIAVVAEQPGSNEAKKALEEILEAAE<br>ELAKSPDPEALRAAVELAEFVVLMQPGSNLAKKALEII<br>LRAAAALANLPDPESRKEADRAAEFVRREQPGSELA<br>VVAAIISAVARMGVKMELHPSGNEVKVVIKGLHIKQQ<br>RQLYRDVREAAKKAGVEVEIEVEGDTVTIVVRGGSW<br>SGLEHHHHHH	C2<br>LHD101A
O32-17_A	TTEDIIEVLKLSLEVQKELAEQLKQLLEKLEQLQRKGQ<br>SDEEVRELLEKIKRLVQLIEELAEVQRKLVEVLKLLQE<br>NDSKELTELIRDVVRSLKELYEKSGDEKLLEIALLVLLL<br>LAKLLVQEGNVEEVRQVLEEVKELYEESGTLEAAELA<br>LDVLERVAEEARERGNLLAVAVAEAEAAELAAEATG<br>DPRVRELARELKRLAEEMAAEVRRDPSSSDVREALE<br>LVAEAIRAAVRALRAALRTGDPEVRELARELVRLAVE<br>AAEEVQRNPSSSDVNEALKLIVLAIEAAVLALEAAERA<br>GDPALRERARRRVRAAVEAAEEVQRNPSSEEVRLW<br>AVLVVAAALASSPDPEARKEADKLADEVRRAVPGSE<br>LAVVAAIISAVARMGVEMELLPSGNEVKVVIRGLHIKQ<br>QRQLYRDVREAAKKAGVEVEIEVEGDTVTIVVRGLE<br>HHHHHH	C2<br>LHD101A
I32-2_A	MLRIEIETENEDMPAVEAMLAALSKKVKEVEVTIRAPE<br>WDEEGVKHLAKAYEALAKNAEYAKLTVEGLHIKQQR<br>QLYRDVRETSKKQGVETEIEVEGDTVTIVVRELEHHH<br>HHH	C2<br>LHD101A
O43-14_A	DIEKLCKEAEELAVQARALAILLRALHPDSQAARDAE<br>LLAEMAELAVELACRLAEKHPNADIAKLCIKAASEAAE<br>AARLAALLALLHPDSQAARDAIRLALLAAAAVALACIL<br>AMLHPNADIAKLCIKAASEAAEAARLAAELAMEHPDS<br>QAARDAIRLATLAAEAVMEACRYAMLHPNAEDAKLLI<br>EAASNMAEAVSLYVEALRTGDPELLRKALEELERAV<br>RLVEEAIKRNPDNDAAVRLAVRLAEALKAVAERLQER<br>AKKTGDPELLKLALRALEVAVRAVELAIKSNPDNDEA<br>VETAVRLARELKKVAEELFKRAEKTGDPELLRLARRA<br>LEVAERAVELALESNPDNEEARETREELERGRRLVR<br>ARELAESPDPEDLKEAVRLAEEVVREQPGSEEAKRA<br>LEVILLAAEELAKLPDPEALKEAVKAAEKVVREQPGS<br>NLAKKALEIILRAAAALANLPDPESRKEADKAADKVR<br>REQPGSELAVVAAIISAVARMGVKMELHPSGNEVKV	C4<br>LHD101A

	VIKGLHIKQQRQLYRDVREAAKKAGVEVEIEVEGDTVTIVVRGLEHHHHHH	
O43-36_10A (O43-9_A)	SEIEKASDELKKLIELAKAIWMLLRANSLLLEATSRGDTQSQEVAELARKVIKKMPNSELAEKALELAEKAAESESTQKQEVAKLALKVVLKEPNSERAEKALELAEKAAESESEVLQVIAKKVLEFGVEMEVHPSGNEVKVVIKGLHIKQQRQLYRDVREAAKKAGVEVEIEVEGDTVTIVVRELEHHHHHH	C4<br>LHD101A
O43-9_B	EEAELAYLLGELAYKLGENRIAIRAYRIALKRDPNNAEAWYNLGNANYNTGDYDEAIEYYQKALELDPNNAEAWYNLGNAYYKQGRLEEAIEYYQKALELDPDNVEALKNLGRALEEKVREVEEEIKENPDNDEAVEEAVRLARELKRVAEKLQELAKKTGDAELLKNALRALEVAVRAVELAIKSNPDNDEAVETAVRLARELAKVAEELIERAKKTGDKELLKLAKRALEVAMRAVSLALKSNPDNEEARRVAAELVLLVIRAAVIEMGVTMEEHRSGNRVKVVIKGLHESQQEQLLEDVLRAAEIAGVRVRIRFKGDTVTIVVEGLEHHHHHH	C3<br>LHD101A
**Network designs**		
T33-182_12A (T33-14A)	EEAELAYLLGELAYKLGENRIAIRAYRIALKRDPNNAEAWYNLGNANTNTGDYDEAIEYYQKALELDPNNAEAWYNLGYAYLATGKLDEAIKYFKKALELDPNNLTAKALLYLAKAAKIALEQGDPETAKTIREAGVEIGKNLKKNKSKEAEKVALYLAEAVLIAAEQGDPETAKTIAEAAKEIAELLEKNQSPEVIKVALAAAAAVLVAAKNGDPEKAKEIAEKAKEAAKELEKDPSPEVAKEKIKEFARLLLEAAAEAASSPDPEARKAAEEGAAVVQKIAPGSELAVVAAIISAVARMGVKMELHPSGNEVKVVIKGLHIKQQRQLYRDVREAAKKAGVEVEIEVEGDTVTIVVRGLEHHHHHH	C3<br>LHD101A
T33-182_1B (O43-5_B)	EEAELAYLLGELAYKLGEYRIAIRAYRIALKRDPNNAEAWYNLGNVYYRNGDYDEAIEYYQKALELDPNNAEAWYNLGFAYGRNGDFDNAIKYFKKALELDPNNFAAKAALLAAEAGDEELIRIIDEAYLKGDKVALAAALAAAAAALAAKEAGDEELIKKAREAAREGDVRRAARVLLEAAVATKDAEEVERAREALRAAEELLEDPELRRYVRQVLVVLTVVRFVKEAGVTMEIHESGREVKVVIKGLHESQQEQLLEAVLRAAEEAGVRVRIRFKGDTVTIVVRGLEHHHHHH	C3<br>LHD101B
O43-5_A (O43-12_A)	KVLLLVAVLNLAANLIAEKTLSLTVLVKVLLKLDEETREEVLELIKELLKTREKLTMTAEVEPEDEEAFELLRELINLAIESVKEATIVVKGLHIKQQRQLYRDVRETSKKQGVETEIEVEGDTVTIVVREGSWSGLEHHHHHH	C4<br>LHD101A

O43-12_B<br>(T33-14B)	SEEKIEKLLEELTASTAELKRATASLRAITEELKKNPSEDALVEHNRAIVEHNAIIVENNRIIATVLLAIVAAIATNEATLAADKAKEAGASEVAKLAKKVLEEAEELAKENDSEEALKVVKAIADAAKAAAEAAREGKTEVAKLALKVLEEAIELAKENRSEEALKVVREIARAALAAAQAAEEGKTEVAKLALKVLEEAIELAKENRSEEALKVVLEIARAALAAAQAAEEGKTEVAKLALKVLEEAIEAEELIERAKKTGDKELLKLAKRALEVAMRAVSLALKSNPDNEEARRVAAELVLLVIRAAVIEMGVTMEEHRSGNRVKVVIKGLHESQQEQLLEDVLRAAEIAGVRVRIRFKGDTVTIVVEGGSWSGLEHHHHHH	C3<br>LHD101B
**3-component assemblies**		
pyr-01_comp1	PELFLQDLRSLVEAARILARLARQRGDEHALERAARWAEQAARQAERLARQARKEGNLELALKALQILVNAAYVLAEIARDRGNEELLEYAARLAEEAARQAIEIWAQAMEEGNQQLRTKAAHIILRAAEVLLEIARDRGNQELLEKAASLVDAVAALQQAAAAILEGDVEKAVRAAQEAVKAAKEAGDNDMLRAVAIAAIRIARKAIEKGDVEVAVKALRVAVEAAKQAGDNELLDRVAIEAIRVALKAVAKGDFEVAEKALEVAREAAKQAGSEELLLIVEAARLIIKAIEEGDPSYLEEAKRKLEEALRLLREGLAVNPEDERAYRLSIEALLLLLALLVELILRGTEIVVEVHINGRKTEIEVQGIGIGQALVILEEIREEIEESGSQEVEVNVHSGGQTWTFNVGLEHHHHHH	C3<br>LHD206A
pyr-01_comp2	SHSFILGNASEEARQLIEEVVEEISRKLGTEVRFEKKDGTLHIEVKNLHDEYAQLIADAIVLILEALRSDDSEAKKVARLALEIVKLLPNSELAREALELAREALKSTDSEALKVVYLALRIVQQLPDTELAREALELAKEAVKSTDQEALKSVYEALQRVQDKPNTEEARESLERAKLEVAVRAVELAIKSNPDNDEAVETAVRLARKLKEAAERAQEEAKKTGDAELLKNALRALEVAIEALLLALQSRPDFSEAVELLERLAEELKKLAELLEERAKETGDPELQKLAERALLLAFLALLAALAGRLGVTMLIHEHGNVVFVVILGLKPEQKLELLRDVHRIAHKLGVTLSITFSGDIVVIAVTVGASEEEKKEVRKIVKLIAELLRKAETEEEAKLIVKAAIELADIAKKAIEAAREGNTDEVREQLQRLLEIVREIGLEAAVEAALEAVARVAIEAARRGNTDAVREALEVALEIARESGTTEAVKLALEVVARVAIEAARRGNTDAVREALRVAEEIARESGTEEAKRLAQEVIKRVADEAKKQGNAEAVILAAKVLVKMQPSTEALRLVLEAVALAANLASKVTDEDKQRKLAKEAKELALEAYKEDPSDLALAALALAIILEVVVKMGVKMEVHISGNTVKVVIKGLHESQQEQLHKAVEEAVQKLGVFVLVSHHGDTVTIQVYGGSWSGLEHHHHHH	C3<br>LHD29A

pyr-01_comp3	TVTFDITNISHEAIDIILYGVLGIAAMEGTEVTFHSERGQLQIEVKNLHEKQKRNIEKLIEAALRAQSPDPEDLREAVRIARELVEELPGTPLAEAAEEVIKTAATKLAESPDPEDKREAVEGLDLVAESALARAQQDLERGDIPEAVQDLVEALNAAVAAGSQDRLDQISEQAERARKLAEKQGDKLLAFVLALISLVAQMGVPVEIIPEGNEVLVVIKGLHSSQQLKLLKLVVKLANKLGVNVHISFRGDTVTIRVRGGSWSGLEHHHHHH	Asym<br>LHD206B, LHD29B
pyr-05_comp1	PELFLQDLRSLVEAARILARLARQRGDEHALERAARWAEQAARQAERLARQARKEGNLELALKALQILVNAAYVLAEIARDRGNEELLEYAARLAEEAARQAIEIWAQAMEEGNQQLRTKAAHIILRAAEVLLEIARDRGNQELLEKAASLVDAVAALQQAAAAILEGDVEKAVRAAQEAVKAAKEAGDNDMLRAVAIAALRIARAALLKGDVEVAVKALRVAVEAAKQAGDNELLRRVAIEAIRVALAALGKGDVEVAERALEVAEEAAKQAGDEGLLAIVRAVRALVRGDIETAEKDLEEAVKLARERGDPELLKEALLVGLFVKLLELLERGTEIVVEVHINGRKTEIEVQGVSLGQLVVILEVIREEIEREGSPEVEVNVHSGGQTWTFNAGLEHHHHHH	C3<br>LHD206A
pyr-05_comp2	SHSFILGNASEEARQLIEEVVEEISRKLGTEVRFEKKDGTLHIEVKNLHDEYAQLIADAIVLILEALRSDDSEAKKVARLALEIVKLLPNSELAREALELAREALKSTDSEALKVVYLALRIVQQLPDTELAREALELAKEAVKSTDQEALKSVYEALQRVQDKPNTEEARESLERAKLEVAVRAVELAIKSNPDNDEAVETAVRLARKLKEAAERAQEEAKKTGDAELLKNALRALEVAIEALLLALQSRPDLEEGRELLERLAEELKKLAELLEERAKETGDPELQKLAERALLLAFLALLAAWAGEFGVTMLIHEHGNVVFVVILGLRPEQKLELALRVHRIAHKLGVTLSITFSGDIVVIAVTVGASEEEKKEVRKIVKLIAELLRKAETEEEAKLIVKAAIELADIAKKAIEAAREGNTDEVREQLQRLLEIVREIGLEAAVEAALEAVARVAIEAARRGNTDAVREALEVALEIARESGTTEAVKLALEVVARVAIEAARRGNTDAVREALRVAEEIARESGTEEAKRLAQEVIKRVADEAKKQGNAEAVILAAKVLVKMQPSTEALRLVLEAVALAANLASKVTDEDKQRKLAKEAKELALEAYKEDPSDLALAALALAIILEVVVKMGVKMEVHISGNTVKVVIKGLHESQQEQLHKAVEEAVQKLGVFVLVSHHGDTVTIQVYGLEHHHHHH	C3<br>LHD29A
pyr-05_comp3	TVTFDITNISHEAIDIILEGVLGIAALEGTEVTFHSERGQLQIEVKNLHEKQKRNIEKLIEAALRAQSPDPEDRREAVRIARELVEEAPGTPLAEAAIRVIITAATKLAESPDPEDKREAVRALRLAAEAAIRAAERAAERGDLEEAIENLRTAQAAAIAAGDVELLRKISEQARRLSKLARKQGDKVLELLIEILRLVAEMGVRITIEPRGDEVTVVVTGLHAGDQLKLLDLVIKIANKLGVNVHISVRGDTVTIRVRGGSWSGLEHHHHHH	Asym<br>LHD206B, LHD29B

pyr-07_comp1	PELFLQDLRSLVEAARILARLARQRGDEHALERAAR<br>WAEQAARQAERLARQARKEGNLELALKALQILVNAA<br>YVLAEIARDRGNEELLEYAARLAEEAARQAIEIWAQA<br>MEEGNQQLRTKAAHIILRAAEVLLEIARDRGNQELLE<br>KAASLVDAVAALQQAAAAILEGDVEKAVEAAQKGQD<br>TASRGGDADMLRAVAIAALRIAEEALKQGDVEVAVEA<br>IEVAQRAASLAGDAELLDRVVEKAERIAEEARKQGNE<br>AVALLAEATALLARATRARATGDAAELERVREEARRL<br>RERAKEIAERARKEGNKEVALEAVLAIIVCEVAIMGAD<br>LVTSPSGDTITVTITGLHIKQQRQLYRLVREGAKLAGV<br>EVEIEVEGDTVTIVVRGLEHHHHHH	C3<br>LHD206A
pyr-07_comp2	SHSFILGNASEEARQLIEEVVEEISRKLGTEVRFEKKD<br>GTLHIEVKNLHDEYAQLIADAIVLILEALRSDDSEAKKV<br>ARLALEIVKLLPNSELAREALELAREALKSTDSEALKV<br>VYLALRIVQQLPDTELAREALELAKEAVKSTDQEALK<br>SVYEALQRVQDKPNTEEARESLERAKLEVAVRAVEL<br>AIKSNPDNDEAVETAVRLARKLKEAAERAQEEAKKT<br>GDAELLKNALRALEVAIEALLLALQSRPDFSEAVELLE<br>RLAEELKKLAELLEERAKETGDPELQKLAERALLLAF<br>LALLAALVAKMGVTMLIHEHGNVVFVVILGLHPEQVL<br>ELAREVIEIAHKLGVTLSITFSGDIVVIAVTVGASEEEK<br>KEVRKIVKLIAELLRKAETEEEAKLIVKAAIELADIAKKA<br>IEAAREGNTDEVREQLQRLLEIVREIGLEAAVEAALEA<br>VARVAIEAARRGNTDAVREALEVALEIARESGTTEAV<br>KLALEVVARVAIEAARRGNTDAVREALRVAEEIARES<br>GTEEAKRLAQEVIKRVADEAKKQGNAEAVILAAKVLV<br>KMQPSTEALRLVLEAVALAANLASKVTDEDKQRKLA<br>KEAKELALEAYKEDPSDLALAALALAIILEVVVKMGVK<br>MEVHISGNTVKVVIKGLHESQQEQLHKAVEEAVQKL<br>GVFVLVSHHGDTVTIQVYGGSWSGLEHHHHHH	C3<br>LHD29A
pyr-07_comp3	TVTFDISDISPEAQLTIARGVVGIAIEEGTEVTFHSERG<br>RLIIEVKNLKEESKERIELLIEASQLVDRARATLEKIKK<br>AAESGDEKALEEAQKEAEEVRKRAEEVREKAKELGD<br>EESERLAEEAYRLALIALLLALIAKLVLKMGVKAEIRFE<br>GDTAVVTITGLHESQQEQLLEFVLRAAEKLGVRVRIR<br>FKGDTAVITARGGSWSGLEHHHHHH	Asym<br>LHD206B,<br>LHD29B
pyr-21_comp1	PELFLQDLRSLVEAARILARLARQRGDEHALERAAR<br>WAEQAARQAERLARQARKEGNLELALKALQILVNAA<br>YVLAEIARDRGNEELLEYAARLAEEAARQAIEIWAQA<br>MEEGNQQLRTKAAHIILRAAEVLLEIARDRGNQELLE<br>KAASLVDAVAALQQAAADILEGNTDRAVESLQRAVK<br>AAKEAGDNDMLRAVAIAALKVADAALAIGDTATASLA<br>VRVATEAAKLAGDEELLRRVEEKAQEIARAAEENKR<br>AAQTIADQADKDGDDGKKAEALLLIAKSLKAIVGVARI<br>SGDKERQEKAKEDLKRLLEEAKKVAQSAKEQGDKD<br>TAIKALLVGIYAIAALMGVDVREEPSGKVVKVVITGIHV<br>KQQRQLYRVVRELAKLAGVEVEIEVEGDQVTIVVRGL	C3<br>LHD206A

	EHHHHHH	
pyr-21_comp2	SHSFILGNASEEARQLIEEVVEEISRKLGTEVRFEKKD<br>GTLHIEVKNLHDEYAQLIADAIVLILEALRSDDSEAKKV<br>ARLALEIVKLLPNSELAREALELAREALKSTDSEALKV<br>VYLALRIVQQLPDTELAREALELAKEAVKSTDQEALK<br>SVYEALQRVQDKPNTEEARESLERAKLEVAVRAVEL<br>AIKSNPDNDEAVETAVRLARKLKEAAERAQEEAKKT<br>GDAELLKNALRALEVAIEALLLALQSRPDFSEAVELLE<br>RLAEELKKLAELLEERAKETGDPELQKLAERALLLAF<br>LALLAALVAKMGVTMLIHEHGNVVFVVILGLHKQQAL<br>QLLRDVHRIAHKLGVTLSITFSGDIVVIAVTVGASEEE<br>KKEVRKIVKLIAELLRKAETEEEAKLIVKAAIELADIAKK<br>AIEAAREGNTDEVREQLQRLLEIVREIGLEAAVEAALE<br>AVARVAIEAARRGNTDAVREALEVALEIARESGTTEA<br>VKLALEVVARVAIEAARRGNTDAVREALRVAEEIARE<br>SGTEEAKRLAQEVIKRVADEAKKQGNAEAVILAAKVL<br>VKMQPSTEALRLVLEAVALAANLASKVTDEDKQRKL<br>AKEAKELALEAYKEDPSDLALAALALAIILEVVVKMGV<br>KMEVHISGNTVKVVIKGLHESQQEQLHKAVEEAVQK<br>LGVFVLVSHHGDTVTIQVYGGSWSGLEHHHHHH	C3<br>LHD29A
pyr-21_comp3	TVTFDITNISPEAQDIILYGVLGIAAMEGTEVTFHSERG<br>QLQIEVKNLHEKQKRNIEKLILAAQLAQSPDPLAAAKA<br>VEIAKELVEELPGTPLATAAKQVILAAAVTQISNLADR<br>FGVTREIIKSGGTVTIVIKGLHESQQEQLLELVLTFAQ<br>RAGIRVKIRFKGDTVTIVIRAGSWSGLEHHHHHH	Asym<br>LHD206B, LHD29B
D32_comp1 (O43-36_B, shared component)	SDEAIKRLLEELRASTAELKRATASLRAITEELKKNPS<br>EDALVEHNRAIVEHNAIIVENNRIIAAVLEAIVAEFILAK<br>ARLLVWEARKKGNPEEVLEAAKKALRVAEEAAKAGI<br>EAIFKAAAEAALEIAMRLIDLATKKGDPDLVREAQKVA<br>ERVAELADKNGDEEVKLKAYAAAVAAEIIRRIETLKRS<br>GSSYEDIRQTLRETLERIIEELKRRGVDSSQIVWAIIYV<br>AVAVMGVTMETHKSGNEVKVVIKGLHESQQEELLEL<br>VLRAAELAGVRVRIRFKGDTVTIVVRGLEHHHHHH	C3<br>LHD101B
D32_comp2	SEKARIAVENLEAALRLRQAALEMLKSAIKIYEDNPSD<br>EKAKRYLELTIKVALMSHRLLLASLELADKALREEGS<br>DDSAEKVRKEALKFLEESLDVLIETANKLGDQKLLSE<br>VLQEAIRVKVVEMGVTMETHKSGNVVKVVIKGLHES<br>QQETLRKKVHELLRKLGVVAVTQKHGDTVTIYVTEG<br>SWSGLEHHHHHH	C2<br>LHD202B
D32_comp3	SVEFHIVNIDEETAQAIEKKVKELSKREGTEVRFEKRD<br>GELTIEVKNLHEEDLQAILDFIEAALKVSKARELVRQA<br>REKGSIEDLVKAAETAKEAVRAQPGSEAAKLALKVIL<br>EAAAELANSPDPESRKLADKYADFVRREQPGSELAV<br>VAAIISAVARMGVKMELHPSGNEVKVVIKGLHIKQQR<br>QLYRDVREAAKKAGVEVEIEVEGDTVTIVVRGGSWS	Asym<br>LHD101B, LHD202A

	GLEHHHHHH	
	**2D layers**	
O43-36_B (shared component)	SDEAIKRLLEELRASTAELKRATASLRAITEELKKNPS<br>EDALVEHNRAIVEHNAIIVENNRIIAAVLEAIVAEFILAK<br>ARLLVWEARKKGNPEEVLEAAKKALRVAEEAAKAGI<br>EAIFKAAAEAALEIAMRLIDLATKKGDPDLVREAQKVA<br>ERVAELADKNGDEEVKLKAYAAAVAAEIIRRIETLKRS<br>GSSYEDIRQTLRETLERIIEELKRRGVDSSQIVWAIIYV<br>AVAVMGVTMETHKSGNEVKVVIKGLHESQQEELLEL<br>VLRAAELAGVRVRIRFKGDTVTIVVRGLEHHHHHH	C3<br>LHD101B
C3-23A linker for *P*3-23 2D array	PELFLQDLRSLVEAARILARLARQRGDEHALERAAR<br>WAEQAARQAERLARQARKEGNLELALKALQILVNAA<br>YVLAEIARDRGNEELLEYAARLAEEAARQAIEIWAQA<br>MEEGNQQLRTKAAHIILRAAEVLLEIARDRGNQELLE<br>KAASLVDAVAALQQAAAAILEGDVEKAVRAAQEAVK<br>AAKEAGDNDMLRAVAIAAAKIAKEAEKAGDEEAREK<br>ALLIAGEALAAIYEQGDYDGAFETLERLAKMFGVTPE<br>EALNRLIEAALKAGDKELVETLVKILVELAKKEGKKTV<br>TITLEKVLPVEQLLYLYEVLVKEAKGMEVVLTAKTHIK<br>QQRQLYRDVRETSKKQGVETEIEVEGDTVTIVVRELE<br>HHHHHH	C3<br>LHD101A
	**3D crystal designs**	
1-component O3 cage (shared component)	PELFLQDLRSLVEAARILARLARQRGDEHALERAAR<br>WAEQAARQAERLARQARKEGNLELALKALQILVNAA<br>YVLAEIARDRGNEELLEYAARLAEEAARQAIEIWAQA<br>MEEGNQQLRTKAAHIILRAAEVLLEIARDRGNQELLE<br>KAASLVDAVAALQQAAAAILEGDVEKAVRAAQEAVK<br>AAKEAGDNDMLRAVAIAAIRIARKAIEKGDVEVAVKAL<br>RVAVEAAKQAGDNDLLIRVAIEAIRVALKAVRKGDFE<br>VAEKALRVAEEAALQAGDEELLLVVLATRLIIEAAREG<br>DSRYLEAAEKLLELALDRTKRRLAEDPNDERARRISV<br>EALLLRLLALLVREILRGTEIVVEVHINGRKTEIEVQGI<br>GIGQALVILEEIREEIEESGSQEVEVNVHSGGQTWTF<br>NVGLEHHHHHH	O3<br>LHD206A
3-component D4 cage	SGPELFLQDLRSLVEAARILARLARQRGDEHALERAA<br>RWAEQAARQAERLARQARKEGNLELALKALQILVNA<br>AYVLAEIARDRGNEELLEYAARLAEEAARQAIEIAAQA<br>MEEGNFELALEALEIINEAARVLARIAHHRGNQELLEK<br>AASLTHASAALSRAIAAILEGDVEKAVRAAQEAVKAA	D4

	KEAGDNDMLRAVAIAALRIAKEAEKQGNVEVAVKAA<br>RVAVEAAKQAGDNELLAKAAEEALRIAEEALKQGNV<br>EVAVKAARVAVEAAKGVKNLKEELLARAAKVFAALA<br>PHLPLEAFELALEALVIALEQGHVDAAEAILEGLEAFL<br>EADPSVTPEQRAALAFAAAALLARYNALGRKEEAER<br>ARERARRLIEELEGSHHHHHH<br><br>SGPRLVLRALENMVRAAHTLAEIARDNGNEEWLERA<br>ARLAEEVARRAERLAREARKEGNLELALKALQILVNA<br>AYVLAEIARDRGNEEELEYAARLAEEAARQAIEIAAQ<br>AMEEGNLELALKALQIIVNAAYVLAEIARDRGNEELLE<br>KAASLAEAAAALAEAIAAILEGDVEKAVRAAQEAVKA<br>AKEAGDNDMLRAVAIAALRIAKEAEKQGNVEVAVKA<br>ARVAVEAAKQAGDNELLRKVAEVALRIAKEAEKQGN<br>VEEAVKALRVAVEAAKQAGDAEILVRVAEQALKLLPQ<br>ANEEELKKLVKVIEEAIKALVKLGDVEGLARLVEQAVE<br>QAEKYAELGRKEELEALAKIGAKAVEELAREIRRREE<br>ERGSHHHHHH<br><br>SGLELALKALQILVNAAYVLAEIARDRGNEELLEKAAR<br>LAEEAARQAERIARQARKEGNLELALKALQILVNAAY<br>VLAEIARDRGNEELLEYAARLAEEAARQAIEIWAQAM<br>EEGNQQLRTKAAHIILRAAEVLLEIARDRGNQELLEKA<br>ASLVDAVAALQQAAAAILEGDVEKAVRAAQEAVKAA<br>KEAGDNDMLRAVAIAALRIARAALLKGDVEVAVKAAQ<br>VAVEAAKQAGDNELLRRAAIEAVRVALAALGKGDKE<br>VALKALKVAEKAAALAGDDGLLAIVLAVRAIAEGDTEL<br>ARLFAEAAARLAKKEGDPELLKEALLVGLLVKLRELL<br>ERGTEIVVEVHINGRKTEIEVQGVSLGQLVVILEVIRE<br>EIEREGSPEVEVNVHSGGQTWTFNAGGSHHHHHH	LHD206A
C3 linker for O3_C3-*F*432-23	PELFLQDLRSLVEAARILARLARQRGDEHALERAAR<br>WAEQAARQAERLARQARKEGNLELALKALQVVVEA<br>ARVLAEIARDRGNEELLELAARLAERAAREAIELARQ<br>AAIEGNEELFEAALEVVLRAAEVLLEIARDRGNQELLE<br>KAASLVDAVAALQQAAAAILEGDVEKAVRAAEEALAA<br>ARAGGDAEMLVLVAVVALRIGEAAAEAGDREAAERA<br>FSIAVQAILEALALVDEERGKEILEEIGPLLVEFAKRNP<br>ELKVEASIKLDDDEEKLELIMELVRELAKHIEVSLKVE<br>VASSIILKAVELACKIAKEAGLKEATLTTKTSKSQQEQ<br>LLKDVLKTANKQGVNVHISFRGDTVTIRVREGSWSG<br>LEHHHHHH	C3<br>LHD206B
C3 linker for O3_C3-*F*432-4	ELLLDVAAAIAYTIALVEARGDEEAIRRILERMQALPLR<br>VRATAVAIYLEYLELRGEDVAPLIRLVTEIDPEVLGEA<br>LRLRAAMSPEPLTLELTFDESEFELALKVIEALRGVKG<br>VHLNITVDASLEQLKKLIEAAVAAAKASGSTITITTTCD<br>KETQKELLKDVLKTANKQGVNVHISFRGDTVTIRVRE<br>GSWSGLEHHHHHH	C3<br>LHD206B

Supplementary Table 4. T33-549 cryoEM data acquisition

Microscope	Krios (NCCAT)
Voltage (kV)	300
Exposure navigation	Image shift
Nominal magnification	105,000x
Detector	Gatan K3
Detector mode	super-resolution
Automation software	Leginon
Unbinned pixel size (Å/pix)	0.4124
No. of frames	50
Dose rate (e-/Å/s)	29.40
Exposure per frame (e-/Å^2)	1.423
Total dose (e-/Å^2)	58.80
Exposure time (s)	2.0
Defocus range (μm)	0.6 - 2.2
No. of micrographs	12,276

Supplementary Table 5. T33-549 cryoEM processing

**Image Processing**	
Software	Cryosparc v.3
No. of final particles	662,340
Box size (pixels)	400
Map's pixel size (Å/pix)	1.236
Symmetry	T
Map resolution (Å):<br>FSC 0.143 (unmasked / masked)	6.4 / 6.1

Map sharpening B factor ($Å^2$)	None applied
Sphericity from 3DFSC (unmasked/masked):	0.972 / 0.964
EMDB ID:	EMD-47128
EMPIAR	EMPIAR-12313
**Coordinate refinement**	
PDB	9DRL
Software	Chimera & Phenix
FSC model vs map = 0.5 / 0.143 (Å)	7.7 / 7.0
No. of residues:<br>● Protein	10,608
Root-mean-square deviation:<br>● Bond length (Å)<br>● Bond angle (°)	0.006<br>0.779
Map correlation coefficient<br>● Map CC (mask)<br>● Map CC (box)<br>● Map CC (peaks)<br>● Map CC (volume)	0.79<br>0.93<br>0.73<br>0.77
Molprobity score	1.88
Molprobity clash score	23.80
Rotamer outliers (%)	0.00
C-beta deviations (%)	0
Ramachandran plot (%):<br>● Favored<br>● Allowed<br>● Outliers	97.96<br>2.04<br>0.00
Rama-Z (Z-score, RMSD):<br>● Whole (N = 10.560)<br>● Helix (N = 9,360)<br>● Sheet (N = 648)<br>● Loop (N = 552)	0.05<br>0.21<br>0.60<br>0.95

CaBLAM outliers (%)	0.11

Supplementary Reference

1. Kibler, R. D. *et al.* Design of pseudosymmetric protein hetero-oligomers. *Nat. Commun.* **15**, 10684 (2024).
